# Membrane Sphingolipids Regulate the Fitness and Antifungal Protein Susceptibility of *Neurospora crassa*

**DOI:** 10.3389/fmicb.2019.00605

**Published:** 2019-04-11

**Authors:** Anna Huber, Gregor Oemer, Nermina Malanovic, Karl Lohner, Laura Kovács, Willi Salvenmoser, Johannes Zschocke, Markus A. Keller, Florentine Marx

**Affiliations:** ^1^Division of Molecular Biology, Biocenter, Medical University of Innsbruck, Innsbruck, Austria; ^2^Division of Human Genetics, Medical University of Innsbruck, Innsbruck, Austria; ^3^Institute of Molecular Biosciences, Biophysics Division, University of Graz, Graz, Austria; ^4^Institute of Zoology, University of Innsbruck, Innsbruck, Austria

**Keywords:** sphingolipids, glucosylceramide, lipidomics, *Neurospora crassa*, antimicrobial proteins, *Penicillium chrysogenum*

## Abstract

The membrane sphingolipid glucosylceramide (GlcCer) plays an important role in fungal fitness and adaptation to most diverse environments. Moreover, reported differences in the structure of GlcCer between fungi, plants and animals render this pathway a promising target for new generation therapeutics. Our knowledge about the GlcCer biosynthesis in fungi is mainly based on investigations of yeasts, whereas this pathway is less well characterized in molds. We therefore performed a detailed lipidomic profiling of GlcCer species present in *Neurospora crassa* and comprehensively show that the deletion of genes encoding enzymes involved in GlcCer biosynthesis affects growth, conidiation and stress response in this model fungus. Importantly, our study evidences that differences in the pathway intermediates and their functional role exist between *N. crassa* and other fungal species. We further investigated the role of GlcCer in the susceptibility of *N. crassa* toward two small cysteine-rich and cationic antimicrobial proteins (AMPs), PAF and PAFB, which originate from the filamentous ascomycete *Penicillium chrysogenum*. The interaction of these AMPs with the fungal plasma membrane is crucial for their antifungal toxicity. We found that GlcCer determines the susceptibility of *N. crassa* toward PAF, but not PAFB. A higher electrostatic affinity of PAFB than PAF to anionic membrane surfaces might explain the difference in their antifungal mode of action.

## Introduction

Fungi are pioneers in colonizing the most diverse habitats with access to organic and inorganic nutrients that enables a saprophytic and parasitic way of life. The barrier function of the plasma membrane is crucial for cellular homeostasis and cell environment interactions. The activity of enzymes, transporters and receptors embedded in and along this lipid bilayer ensures important vital functions, such as cell wall synthesis, growth, cell polarity establishment, reproduction and optimal adaptation to environmental changes (Los and Murata, 2004; Fontaine, 2017). As such, any alteration of the lipid composition of the plasma membrane might impact the distribution, regulation, activity and signaling function of membrane proteins, with adverse effects on the fungal cell fitness.

Our knowledge on GlcCer biosynthesis in fungi is based on investigations on yeasts, such as the human pathogenic *Cryptococcus neoformans* (Rhome et al., 2007; Munshi et al., 2018) and *Candida albicans* (Oura and Kajiwara, 2010) and on some few reports on filamentous ascomycetes, such as the human pathogen *Scedosporium apiospermium* (Rollin-Pinheiro et al., 2014), the model organisms *Neurospora crassa* (Park et al., 2005) and *Aspergillus nidulans* (Fernandes et al., 2016), the plant pathogen *Fusarium graminearum* (Ramamoorthy et al., 2007, 2009) and the post-harvest pathogen *Penicillium digitatum* (Zhu et al., 2014). Notably, *Saccharomyces cerevisiae* and some other yeast species lack the GlcCer synthesis gene and therefore, membranes do not contain this sphingolipid (Saito et al., 2006).

Glucosylceramide backbones of fungal origin show a characteristic consensus structure with less structural variations than GlcCer from plants. However, genus- and species-specific modifications of the sphingoid base and the fatty acid chains exist in this fungal lipid group (Warnecke and Heinz, 2003). Fungal GlcCer is a sugar-bound sphingolipid, composed of a long chain (C_18_) sphingoid base (methyl-branched and diunsaturated) linked to a long chain (C_16_ or C_18_) hydroxy fatty acid and a glucose moiety (Del Poeta et al., 2014).

Glucosylceramide is located mostly in the outer leaflet of the plasma membrane with the carbohydrate group exposed to the extracellular space and associates with sterols and proteins to form so called lipid rafts. These are microdomains that influence the membrane fluidity and regulate the organization and integrity of the membrane (Alvarez et al., 2007; Singh et al., 2012; Guimarães et al., 2014). Furthermore, intermediate compounds of the GlcCer pathway, e.g., sphingosine, ceramide and phosphorylated derivatives thereof, are involved in cell signaling related to cell growth and apoptosis (Ohanian and Ohanian, 2001).

As such, the GlcCer synthesis pathway has attracted increasing attention due to its reported role in fungal pathogenicity, growth, morphology, cell polarity establishment and alkali tolerance (Del Poeta et al., 2014; Guimarães et al., 2014). Differences in the structure of GlcCer between fungi, plants and animals/humans and a different set of key enzymes involved in the fungal GlcCer synthesis render this pathway a promising target for new generation therapeutics (Warnecke and Heinz, 2003; Nimrichter and Rodrigues, 2011; Del Poeta et al., 2014; Pan et al., 2018).

Small cysteine-stabilized and cationic AMPs are exceptionally interesting candidates for developing novel treatment strategies. Numerous studies generated detailed insight into the fast-growing group of fungal AMPs in respect to their specificity, activity, structure and function (Garrigues et al., 2016; Paege et al., 2016; Sonderegger et al., 2018; Tóth et al., 2018). We have been extensively studying the function and the structural properties of two AMPs, PAF and PAFB, that are secreted from the penicillin producer *Penicillium chrysogenum* (Marx et al., 2008; Fizil et al., 2015; Sonderegger et al., 2017; Huber et al., 2018). Importantly, their antifungal activity is closely linked with their internalization (Huber et al., 2018) and PAF triggers a rapid and sustained increase of the cytoplasmic calcium concentration in *N. crassa* (Binder et al., 2010). A detailed knowledge of the mode of action of AMPs and their way to interact with sensitive target fungi is supportive for their future exploitation.

In this study, we provide for the first time a comprehensive qualitative and quantitative analysis of sphingolipid species present in the plasma membrane of *N. crassa*. The availability of a gene deletion library of this model fungus prompted us to dissect in detail the GlcCer biosynthesis pathway using mutant strains defective in the distinct enzymatic steps. Phenotypic analysis evidenced that the intermediates of this pathway play an important role in growth, morphology, conidiogenesis and stress response in *N. crassa*. We further show that the GlcCer biosynthesis pathway mediates PAF but not PAFB susceptibility of the highly sensitive *N. crassa*, which suggests that the mode of action differs considerably between these two AMPs. This can be explained by the fact that membrane binding of both AMPs is electrostatically driven revealing a higher affinity of PAFB than PAF to anionic membrane surfaces, which at least in part may compensate the GlcCer depletion in the respective mutants.

## Materials and Methods

### Strains, Media and Cultivation Conditions

All strains and media used in this study are listed in Table 1 and Supplementary Data Sheet S1, Supplementary Table S1.

**Table 1:** Fungal strains used in this study.

Strain	Genotype	Gene/strain identification no.	Source
*N. crassa wt*	*ORS-SL6a (mat a)*	FGSC4200	FGSC
*N. crassa wt*	*74-OR23-1VA (mat A)*	FGSC2489	FGSC
*N. crassa*Δ*ku70*	*mus-51::bar*^+^*; mat a*	FGSC9718	FGSC
*N. crassa* Δ*lac1*	*lac::hph^r^; mat a*	NCU02468, FGSC13903	FGSC
*N. crassa* Δ*des*-1	*des-1::hph^r^; mat A*	NCU08927, FGSC15707	FGSC
*N. crassa* Δ*des-2*	*des-2::hph^r^; mat a*	NCU02408, FGSC16221	FGSC
*N. crassa* Δ*smt*	*smt::hph^r^; mus-51::bar*^+^*; mat a*	NCU07859, FGSC13992	FGSC
*N. crassa* Δ*gcs*	*gcs::hph^r^; mat A*	NCU01116, FGSC13794	FGSC
*P. chrysogenum paf*	OE*paf*; *nat1^r∗^*	NCBI, Pc24g00380	Sonderegger et al., 2016
*P. chrysogenum pafB*	OE*pafB*; *nat1^r∗^*	NCBI, Pc12g08290	Huber et al., 2018

*^∗^OE, overexpression strains, carrying multiple integreations of *paf* and *pafB* expression cassettes, respectively; *nat1*^*r*^, nourseothricin resistance gene.*

The strains *P. chrysogenum paf* and *P. chrysogenum pafB* were grown on solid minimal medium (MM) at 25°C for 48–72 h for conidia generation and in liquid MM at 25°C for 72 h for AMP production (Huber et al., 2018). All *N. crassa* strains were grown on Vogel’s agar [supplemented with 200 μg/mL hygromycin or 400 μg/mL phosphinothricin (PT)] at 37°C under continuous light for 48–72 h to generate conidia. Antibiotics were omitted from experiments performed for phenotype analysis. For all experiments, conidia were freshly harvested in spore buffer [0.9% NaCl (w/v), 0.01% Tween 80 (v/v)].

Fungal biomass of the *N. crassa* strains for dry weight determination and membrane lipid extraction was generated by cultivating 2.5 × 10^4^ conidia in 50 mL liquid Vogel’s medium for 72 h at 25°C under continuous shaking.

Colony formation and growth of *N. crassa* strains was monitored on Vogel’s agar in petri dishes (VWR) after 72 h of incubation at 25°C. After 24 h of cultivation, the diameter of the colonies was determined.

For quantification of conidia production, a defined number of spores from each strain was plated in 6-well plates (VWR) on 4 mL Vogel’s agar/well and spores were harvested after 72 h of incubation at 25°C.

The growth inhibition assays and stress-tests with the *N. crassa* strains were performed in 24-well plates (VWR) on Vogel’s agar with the respective supplementations. Plates were incubated at 25°C for 72 h.

For the broth microdilution assays, the determination of germination efficiency and germ tube length as well as for protein uptake studies, the *N. crassa* strains were grown in 0.2 × Vogel’s medium in 96-well plates (VWR) at 25°C for the indicated time points.

### Membrane Lipid Extraction

Freeze dried mycelia of the *N. crassa* strains were crashed for 1 min at 20 Hz using a Mixermill (MM400, Retsch). The total protein content of each sample was determined by Bradford protein assay (Bio-Rad), after dissolving the sample in dH_2_O.

Subsequently lipids were extracted from biomass corresponding to 500 μg total protein by adding 500 μL chloroform:methanol (2:1) containing 0.5 μM of the internal standards (tetramyristoylcardiolipin and N-heptadecanoyl-D-*erythro*-sphingosine [(C17 ceramide), Avanti Polar Lipids]. Mycelia were further homogenized 4 × 30 sec at 20 Hz and then incubated for 5 min in an ultrasonic bath. For phase separation, samples were centrifuged for 10 min at 14,000 rpm (4°C) and the organic phases were transferred to glass tubes. This chloroform:methanol extraction step was repeated once more. Combined organic phases were dried overnight and stored at -20°C until analysis.

### HPLC-MS/MS Lipidomics

For lipid identification and quantification, lipid extracts of five biological replicates of each strain were used for analysis. Details about equipment and parameters used for lipidomics are provided in Supplementary Data Sheet S1, Supplementary Table S2. The detection of phospholipids and sphingolipids was performed according to a method adapted from Oemer et al. (2018). Briefly, lipid extracts were dissolved in 100 μL mobile phase and 10 μL were injected into an Ultimate 3000 HPLC system (Dionex). Chromatographic separation of analytes was achieved by using gradient elution as indicated (Supplementary Data Sheet S1, Supplementary Table S2). Lipid components were detected with an online coupled LTQ Velos mass spectrometer (Thermo Fisher Scientific) operated in positive ESI mode and within a range of 460–1650 m/z. Raw data were converted into the mzML open format and analyzed with MZmine2 (V2.30) (Pluskal et al., 2010). A manually curated peaklist (Supplementary Data Sheet S1, Supplementary Table S3) was used for targeted peak extraction on basis of cropped (1–22 min, 460–1650 m/z) and baseline corrected data (Parameters: TIC, 0.2 m/z bin with, asymmetric baseline corrector: 10^9^ smoothing, 0.1 asymmetry factor, Rserve). Integrated peaks were further analyzed in R (R Development Core Team, 2015). Lipids were quantified according to their class by linear regression using an external standard dilution series, including three ceramide standards, dihydro-ceramide (dhCer:35:0), ceramide (Cer:36:1) and GlcCer (GlcCer:34:1) and two phospholipids, phosphatidylethanolamine (PE:28:0) and phosphatidylcholine (PC:28:0) and then normalized via the internal standard dihydro-ceramide (18:0/17:0). Absolute quantities were referred to the total protein amount measured in each sample. Please note that the nomenclature of lipid species in this manuscript also reflects the level of their structural characterization by tandem MS/MS in accordance to Liebisch et al. (2013).

### Determination of Conidiation and Colony Establishment

To quantify conidia production, 15 μL aliquots of strain specific spore suspension (2 × 10^4^ per mL) were plated on Vogel’s agar in 6-well plates (VWR). The spores were harvested from the colonies after 72 h of incubation at 25°C and counted. Uni- and multinuclear conidia were quantified by fluorescence microscopy after fluorescence staining with 5 μM of the nuclei-specific dye Hoechst-33342 (Thermo Fisher Scientific) for 5 min at room temperature. One hundred conidia were analyzed for spore type discrimination. The germination efficiency and germ tube length of 6 h old *N. crassa* germlings was determined as described (Sonderegger et al., 2017). For the examination of conidial anastomosis tubes (CAT) fusion, 100 μL of a conidia suspension (5 × 10^5^ per mL) were cultivated in 0.2 × Vogel’s medium for 6 h at 25°C before microscopic validation. Experiments were done in duplicates and repeated at least twice.

### Investigation of Stress Response

Five μL spore suspension (2 × 10^4^ – 2 × 10^5^ spores/mL) were point inoculated in 24-well plates (VWR) and cultivated for 72 h at 25°C on 500 μL Vogel’s agar/well, supplemented with inducers of ROS: hydrogen peroxide (H_2_O_2_, 0–3.5 mM, hydroxyl radicals), menadione (0–0.06 mM; superoxide radicals), *tert*-butylhydroperoxide (*t*-BOOH, 0–1.75 mM; organic hydroperoxide). Experiments were done in duplicates and repeated at least twice.

### Susceptibility Testing With PAF and PAFB

The antifungal proteins PAF and PAFB were produced and purified from *P. chrysogenum* cell-free supernatant as described earlier (Huber et al., 2018). Susceptibility tests were performed in 24-well plates (VWR) with 500 μL Vogel’s agar/well, supplemented with increasing concentrations of PAF or PAFB (0–16 μM), respectively. Five μL of a 2 × 10^5^/mL spore suspension were dotted on the agar and the plates were incubated at 25°C. Images were taken after 72 h, as described below.

Broth microdilution assays were performed in 96-well microtiter plates (Thermo Fisher Scientific) using 0.2 × Vogel’s medium as recently described (Huber et al., 2018). The growth was determined spectrophotometrically by measuring the optical density at 620 nm (OD_620_) with a Fluostar Omega microplate reader (BMG Labtech). The MIC was defined as the protein concentration that inhibited growth by ≥90% compared to the untreated control which was set to be 100%.

The germination efficiency and germ tube length of 6 h old *N. crassa* conidia in the presence of 1 × MIC PAF (0.06 μM) or PAFB (0.25 μM) was determined as described previously (Sonderegger et al., 2017).

All experiments were done in duplicates and repeated at least twice.

### Protein Uptake Studies

Protein labeling using the green fluorophore BODIPY (BP; Life Technologies) was performed as described (Sonderegger et al., 2017). To study the protein uptake in the *N. crassa* strains, 8 μM BP-labeled PAF or PAFB was added to 70 μL conidia (5 × 10^5^ conidia/mL) in 0.2 × Vogel’s medium and incubated for 12 h at 25°C. To monitor the viability of the fungal cells, co-staining with propidium iodide (5 μg/mL) for 10 min at room temperature was performed. Experiments were done in duplicates and repeated at least twice.

### Microscopy and Imaging

The validation of germination efficiency, colony establishment, CAT fusion and AMP susceptibility testing was done with an inverted Leica DM IL LED microscope (Leica Microsystems) and imaging was performed with an AxioCam MR3 camera (Carl Zeiss GmbH). This microscope and imaging system was also used to visualize the edge of colonies grown on solid medium (Supplementary Data Sheet S1, Supplementary Methods). To monitor fungal growth on solid medium, images of samples were taken with a Nikon D5100 SLR camera or a Zeiss DSM950 scanning electron microscope (Carl Zeiss GmbH) (Supplementary Data Sheet S1, Supplementary Methods). For fluorescence microscopy, an Axioplan microscope (Carl Zeiss GmbH), equipped with an AxioCam 503 monochrome camera (excitation/emission filters 365/420 nm for blue fluorescence, 500/535 nm for green fluorescence, 546/590 or 565/620 nm for red fluorescence, Carl Zeiss GmbH) was used for imaging.

Image processing and editing was achieved with the programs ZEN (Carl Zeiss GmbH), Axio Vision software (Carl Zeiss GmbH), GIMP (GNU Image Manipulation Program, version 2.8.20^1^) and Microsoft Power Point (Microsoft Corp.).

### Zeta-Potential Measurements

Large unilamellar vesicles (LUVs) were prepared from isolated lipid extracts from *wt*, Δ*lac*, and Δ*gcs*, respectively, as described (Supplementary Data Sheet S1, Supplementary Methods). Lipids dissolved in chloroform:methanol (2:1, v/v) were evaporated under a stream of nitrogen. After removal of any residual traces of solvent in vacuum overnight, the dried lipid films were hydrated in HEPES buffer at 30°C by intermittent vigorous vortexing. LUVs were obtained by extrusion of the lipid dispersions through a polycarbonate filter (Millipore-IsoporeTM) of 100 nm pore size resulting in LUVs of 100–110 nm in diameter as determined by Zetasizer.

Zeta-potential measurements were performed using the Zetasizer NANO (Malvern Instruments) as described previously (Freire et al., 2011). Briefly, 50 μM of LUVs mixed with defined aliquots of PAF and PAFB, respectively, were injected into the Zeta capillary cell DTS1070 (Malvern Instruments) and equilibrated before measurements for 120 s at 25°C at a constant voltage of 40 V. All samples were prepared in HEPES buffer (10 mM HEPES, 10 mM NaCl, pH 6.8, filtrated through 0.02 μm). Data analysis was processed using the instrumental Malvern’s DTS software to obtain the mean Zeta-potential value calculated from the means of 30 measurements in two experimental repetitions.

### Statistical Analysis

If not otherwise stated, statistical analysis was performed using Microsoft Excel 2010 software (Microsoft Corp.). A two-sample *t*-test with equal variance and one-tailed distribution was applied.

## Results

### Gene Identification and Verification of Respective Gene Deletion Mutants

**FIGURE 1:**
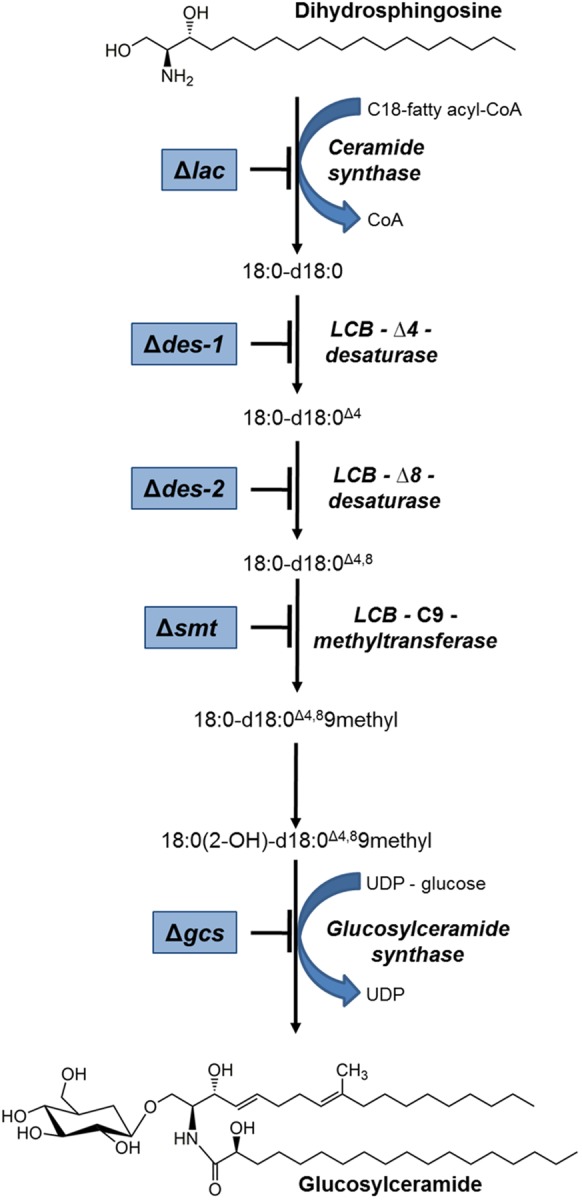
Schematic overview of the GlcCer pathway in *N. crassa*. Mutants that were used in this study are framed in blue and their defective enzyme function are indicated. Structures were plotted with ChemBioDraw (ChemBioOffice).

Gene candidates involved in the GlcCer biosynthesis were identified in the *N. crassa* genome^2^ using the respective *C. albicans* genes as queries^3^. All genes investigated in this study and coding for enzymes of the GlcCer pathway in *C. albicans* were identified in the *N. crassa* genome by bidirectional NCBI-blast analysis^4^ (Table 2): NCU02468 (*lac*) coding for the longevity-assurance protein (ceramide synthase activity), NCU08927 (*des-1*) encoding the dihydroceramide Δ4-desaturase (Plesofsky et al., 2008), NCU02408 (*des-2*) encoding the Δ8-sphingolipid desaturase, NCU07859 (*smt*) encoding the C9-methyltransferase (Ramamoorthy et al., 2009) and NCU01116 (*gcs*) coding for the GlcCer synthase (Plesofsky et al., 2008). The predicted protein functions were verified with UniProt^5^. Generally, protein identities and similarities were >30% and >50%, respectively. The open reading frames (ORFs) of the *N. crassa* genes were analyzed for intron/exon structure, chromosome localization and gene product length. A summary is given in Table 2 and Supplementary Data Sheet S1, Supplementary Table S4. A proposed scheme of the *N. crassa* GlcCer pathway is presented in Figure 1.

**Table 2:** Comparison of enzymes involved in GlcCer synthesis of *C. albicans* and *N. crassa^∗^.*

Function	Organism	Gene name and gene ID	Protein name	No. amino acids (aa) and protein mass (kDa)	Protein identity and similarity	Source
Ceramide synthase	*C. albicans*	LAC1	Lac1p	427 aa 50.24 kDa	39% 59% (*e*-value = 4e-73)	Cheon et al., 2012
	*N. crassa*	NCU02468 Here named: Δ*lac*	Longevity-assurance protein	509 aa 57.98 kDa		This study
LCB-Δ4-desaturase	*C. albicans*	DES1	Sphingolipid delta(4)-desaturase	370 aa 43.39 kDa	57% 69% (*e*-value = 3e-121)	Oura and Kajiwara, 2010
	*N. crassa*	NCU08927 Here named: Δ*des-1*	Dihydroceramide delta(4)-desaturase	406 aa 46.00 kDa		Plesofsky et al., 2008
LCB-Δ8-desaturase	*C. albicans*	SLD1	Delta 8-(E)-sphingolipid desaturase	584 aa 67.38 kDa	46% 61% (*e*-value = 2e-180)	Oura and Kajiwara, 2010
	*N. crassa*	NCU02408 Here named: Δ*des-2*	Fatty acid desaturase	625 aa 71.38 kDa		This study
LCB-C9-methyl-transferase	*C. albicans*	MTS1	Sphingolipid C9-methyltransferase	513 aa 58.77 kDa	60% 73% (*e*-value = 0.0)	Oura and Kajiwara, 2010
	*N. crassa*	NCU07859 Here named: Δ*smt*	Cyclopropane-fatty-acyl-phospholipid synthase	525 aa 59.79 kDa		Ramamoorthy et al., 2009
Glucosylceramide synthase	*C. albicans*	HSX11	Ceramide glucosyltransferase	544 aa 62.75 kDa	34%52% (*e*-value = 3e-92)	Oura and Kajiwara, 2010
	*N. crassa*	NCU01116 Here named: Δ*gcs*	Ceramide glucosyltransferase	546 aa 60.06 kDa		Plesofsky et al., 2008

*^∗^NCBI-blast (https://blast.ncbi.nlm.nih.gov/Blast.cgi), UniProt (http://www.uniprot.org/) and ExPASy-tool (https://web.expasy.org/protparam/) were used for identification and analysis.*

To analyze the membrane lipids and correlate the distinct lipid patterns with the overall fitness of *N. crassa*, we took advantage of the genome-wide deletion library of this model fungus to analyze mutant strains with deleted genes that code for the enzymes involved in the GlcCer biosynthesis: Δ*lac*, Δ*des-1*, Δ*des-2*, Δ*smt*, and Δ*gcs* (Dunlap et al., 2007). These genes were deleted by replacement with the *hygR* cassette (Colot et al., 2006; Park et al., 2011). Respective reference strains were the wildtype (*wt*) strain mating-type *mat a* [isogenic strain ORS-SL6a (FGSC4200) for Δ*lac*, Δ*des-2*, and Δ*smt*] and *mat A* [isogenic strain 74-OR23-1VA (FGSC2489) for Δ*des-1* and Δ*gcs*] (Table 1). The replacement of the respective genes by the *hygR* cassette was verified in all mutants with PCR (Supplementary Data Sheet S1, Supplementary Methods, Supplementary Figure S1, Supplementary Table S5). All mutants were homokaryons, except for Δ*smt*, which exists only as heterokaryon. The genetic background of the Δ*smt* was confirmed by analysis of *smt* gene expression and growth in the presence of PT and hygromycin (Supplementary Data Sheet S1, Supplementary Figure S2). The existence of Δ*smt* as heterokaryon suggests that the C9-methyltransferase is an essential enzyme for *N. crassa* viability, similar to the situation reported for the unicellular fungi *P. pastoris* and *C. neoformans* (Ternes et al., 2006; Ramamoorthy et al., 2009), but contrasting with *A. nidulans* and *F. graminearum*, which possess two sphingolipid C9-methyltransferases, which are suggested to exhibit redundant enzymatic functions (Ramamoorthy et al., 2009; Fernandes et al., 2016). For completeness, we want to mention here that we have also tested the *N. crassa* NCU00008 strain (FGSC13263), which is defective in *gsl-4*, a second ceramide synthase encoding gene present in the genome of this model fungus (Case et al., 2014). The gene *gsl-4* is a *lac-1* paralog and ortholog of the gene *LAG1* from *Saccharomyces cerevisiae* (Schorling et al., 2001) and *lagA* from *Aspergillus* sp. (Cheng et al., 2001; Fontaine, 2017), and is implicated in the inositol phosphorylceramide (IPC) synthesis pathway. But since this mutant showed no difference in its phenotype compared to the *wt* in respect to vegetative growth, conidiation and susceptibility to AMPs (data not shown), we decided to focus in this study on the characterization of the mutants of the GlcCer biosynthesis pathway rather than the IPC pathway.

### Impaired Lipid Composition in *N. crassa* GlcCer Mutants

**FIGURE 2:**
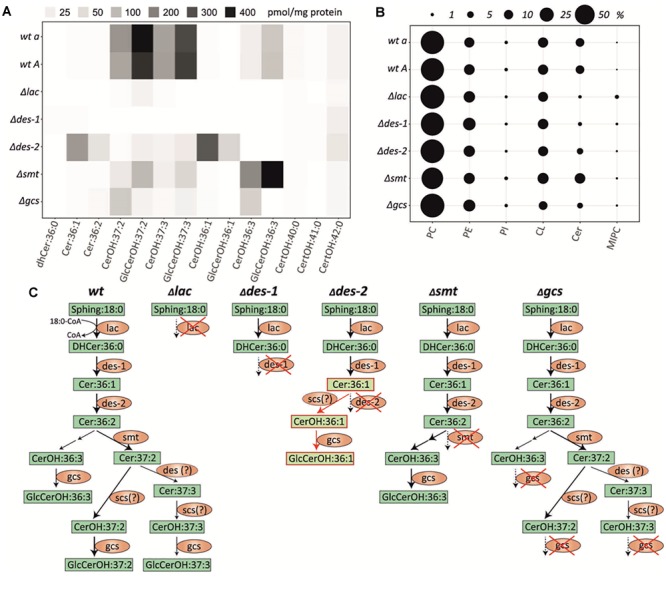
Lipid quantification in the *N. crassa* GlcCer knockout mutants and wildtype strains. **(A)** Heatmap of individual quantifiable ceramide species (means of *n* = 5, for values see Supplementary Data Sheet S1, Supplementary Table S3). **(B)** Total abundance of quantified lipid classes in each strain, respectively. PC, phosphatidylcholin; PE, phosphatidylethanolamine; PI, phosphatidylinositol; CL, cardiolipin; Cer, ceramide; MIPC, mannosylinositol phosphorylceramide. **(C)** Schematic depiction of the ceramide synthesis pathway in *N. crassa wt* and GlcCer mutants Δ*lac*, Δ*des-1*, Δ*des-2*, Δ*smt*, Δ*gcs* based on the results shown in **(A)**. Fatty acid hydroxylation of ceramides by the putative *C. albicans scs7* orthologous gene product (NCU03492) of *N. crassa*. A respective *N. crassa scs* deletion mutant was not included in this study. Green box, lipid species; Orange ellipse, genes/enzymes; Red framed light green box, lipids from alternative pathway; Red cross, respective gene knock-out; Question mark: putative enzymatic step.

To characterize the lipidomic phenotypes caused by the deletion of individual genes involved in GlcCer biosynthesis in *N. crassa*, we performed liquid chromatography (LC-) MS/MS lipid analysis with a special focus on ceramides and glycosphingolipids (putative chemical structures are shown in Supplementary Data Sheet S1, Supplementary Figure S3). Additionally, abundant features related to lipid classes including PC, PE, phosphatidylinositol (PI) and cardiolipin (CL) were also covered (Figure 2 and Supplementary Data Sheet S1, Supplementary Figure S4, Supplementary Table S3). This analysis revealed a clear knock-out specific alteration of the ceramide and GlcCer state of cells, allowing to verify all mutant strains, respectively (Figure 2A). Notably, total levels of unrelated phospholipids (including PC, PE, PI and CL) and their respective species compositions remained unchanged (Figure 2B). The quantification of the lipids can be found in Supplementary Table S1.

Both *wt* strains (FGSC4200 and FGSC2489) were able to generate GlcCerOH:37:2, GlcCerOH:37:3, CerOH:37:2, and CerOH:37:3 (Figure 2C). These ceramide species were already identified in other fungal species, as reported by Leipelt et al. (2001).

Furthermore, non-hydroxylated ceramide levels were below the limit of detection in the *wt*. In most cases the deletion of GlcCer biosynthetic genes resulted in blockage of the pathway resulting in the absence of downstream products. For example, the missing ceramide synthase function in the Δ*lac* knockout caused a complete deficiency of all pathway specific ceramides and GlcCer species. At the same time the phytoCeramide pathway, that utilizes hydroxylated very-long chain fatty acids (C22:0-C24:0) remained unaffected, but further downstream the mannosylinositol phosphorylceramide (MIPC) were accumulated (Figure 2B and Supplementary Data Sheet S1, Supplementary Figure S4). A very similar phenotype was found in the LCB-Δ4-desaturase mutant (Δ*des-1*), where the subsequent Δ4-desaturation step is blocked. However, we found no alteration of the MIPC pathway and no accumulation of its dihydro-ceramide precursor in this mutant. In contrast, blockage of the Δ8-desaturation step in the LCB-Δ8-desaturase mutant (Δ*des-2*) caused an accumulation of its direct precursor Cer:36:1 and enabled flux into a second alternative metabolic route by hydroxylation – but not methylation – and subsequent glycosylation to form GlcCerOH:36:1, which was undetectable in the *wt* strains (Figure 2A). Additionally, small amounts of the regular GlcCerOH:37:2 and GlcCerOH:37:3 products were detected that could potentially be generated by an unspecific desaturase activity. Interestingly, the missing methyltransferase activity in the LCB-C9-methyltransferase knockout (Δ*smt*) caused a strong diversion into the GlcCerOH:36:3 forming pathway, which we found only slightly active in the *wt*. Finally, the GlcCer synthase mutant (Δ*gcs*) almost completely prevented the glycosylation of the CerOH species detected in this study, suggesting a broad recognition of hydroxylated, but not necessarily methylated, ceramides in this step. We further analyzed the mass spectrometric data for the presence of potentially relevant ceramide species reported elsewhere (Costantino et al., 2011; Tian et al., 2015; Singh et al., 2017), but could not detect any additional species.

In summary, while all mutants could be characterized by their specific lipid phenotype, none of them was able to generate the final GlcCer:OH37:2 and GlcCer:OH37:3 products in significant amounts compared to the *wt* strains. The quantity and composition of most other lipid classes including phytoCeramide predominantly Cer[t18:0/24:0(2OH)] was not significantly altered in any mutant (Supplementary Data Sheet S1, Supplementary Figure S4), however, MIPC were elevated in the Δ*lac* mutant.

### Phenotypical Characterization of GlcCer Depletion Mutants

Both *wt* strains (FGSC4200 and FGSC2489) were included as controls in all experiments described below. No difference in the phenotype between these reference strains could be observed. For simplicity reasons, we therefore refer in the following to *wt* as control/reference strain.

### GlcCer Depletion Affects Growth, Asexual Development and Colony Establishment

**FIGURE 3:**
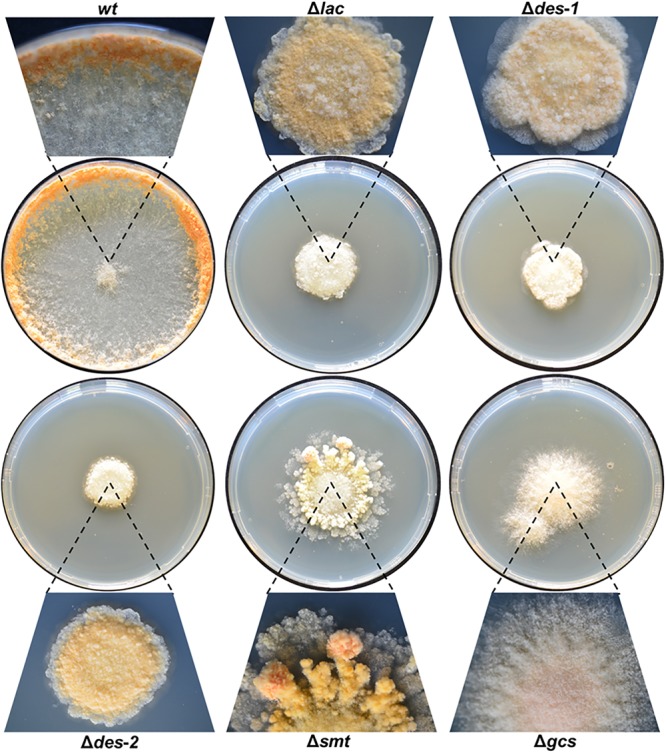
Effect of GlcCer depletion on colony formation of *N. crassa*. Conidia (10^3^) were point inoculated on Vogel’s agar and grown for 72 h at 25°C. Magnifications of the colonies are presented for each strain.

We first studied the effect of depletion of intermediates of the GlcCer synthesis pathway on *N. crassa* strains grown on solid medium (Figure 3). While the reference strain colonized the whole surface, the mutant strains showed disturbed growth with limited radial expansion (Table 3), resulting in small, compact, sharp-edged colonies, except for the mutants Δ*smt* and Δ*gcs*, which had a frayed appearance (Figure 3). Microscopy of the colony edges revealed a hyperbranched morphology of all mutants compared to the *wt* (Supplementary Data Sheet S1, Supplementary Figure S5).

**Table 3:** Effect of GlcCer depletion on the radial growth of *N. crassa* on solid medium.

Strain	Colony diameter [mm]^a,b^
*wt*	22.0 ± 2.0
Δ*lac*	11.7 ± 0.6^∗∗^
Δ*des-1*	11.0 ± 1.0^∗∗∗^
Δ*des-2*	10.8 ± 0.8^∗∗∗^
Δ*smt*	15.7 ± 0.6^∗∗^
Δ*gcs*	6.7 ± 0.6^∗∗∗^

*^*a*^Samples were grown for 24 h at 25°C. ^*b*^Values are given as mean ± SD (*n* = 3). Significant differences (*p*-values) between values were determined by comparison with the *wt* strain. ^∗∗^*p* ≤ 0.005, ^∗∗∗^*p* ≤ 0.0005.*

**FIGURE 4:**
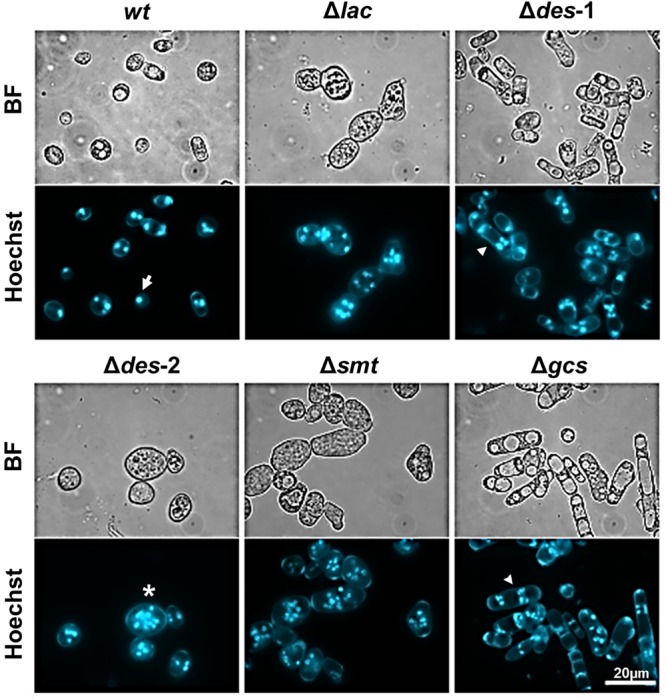
Effect of GlcCer depletion on the formation of uninuclear and multinuclear conidia. Samples were stained for 5 min with Hoechst 33342 before imaging. The white arrow shows an example for uninuclear conidia, the asterisk indicates an example for multinuclear blastoconidia and the arrowhead for multinuclear arthroconidia. BF, Brightfield. Scale bar 20 μm.

The conidiation efficiency was significantly reduced in the mutants Δ*lac*, Δ*des-1*, Δ*des-2*, and Δ*smt* in contrast to the Δ*gcs* strain that generated a similar amount of conidia compared to the *wt* strain (Table 4 and Supplementary Data Sheet S1, Supplementary Figure S6). Importantly, *N. crassa* produces two different types of asexual reproductive organs: the uninucleate microconidia and the multinucleate macroconidia (Springer and Yanofsky, 1989; Bistis et al., 2003). When analyzing the conidia types we observed a prominent shift from 33% microconidia and 67% macroconidia in the reference strain toward more than 90% macroconidia in the mutant strains (Table 4). Among these macroconidia, the mutants Δ*des-*1 and Δ*gcs* produced predominantly arthroconidia, whereas mostly blastoconidia were found in the *wt* and the other mutants (Figure 4).

**Table 4:** The effect of GlcCer depletion on the conidiation of *N. crassa.*

Strain	Conidia per well	Uninuclear conidia [%]^a^	Multinuclear conidia [%]^a^
*wt*	1.1 × 10^8^ ± 2.9 × 10^7^	33.0 ± 7.3	67.0 ± 7.3
Δ*lac*	2.1 × 10^4^ ± 5.8 × 10^3∗∗^	5.2 ± 3.9^∗∗∗^	94.8 ± 3.9^∗∗∗^
Δ*des-1*	1.8 × 10^5^ ± 5.7 × 10^3∗∗^	3.0 ± 1.0^∗∗^	97.0 ± 1.0^∗∗^
Δ*des-2*	1.3 × 10^4^± 2.6 × 10^3∗∗∗^	6.6 ± 2.2^∗∗^	93.5 ± 2.2^∗∗^
Δ*smt*	1.4 × 10^7^ ± 2.1 × 10^6∗^	5.1 ± 4.6^∗∗^	94.9 ± 4.6^∗∗^
Δ*gcs*	1.2 × 10^8^ ± 5.4 × 10^7^	9.1 ± 8.2^∗∗^	90.9 ± 8.2^∗∗^

*^*a*^The number of uninuclear or multinuclear conidia is indicated in (%) compared to the total conidial count of the respective strain, which was set to be 100%. Values are given as mean ± SD (*n* = 3). Significant differences (*p*-values) between values were determined by comparison with the reference strain (*wt*). ^∗^*p* ≤ 0.05, ^∗∗^*p* ≤ 0.005, ^∗∗∗^*p* ≤ 0.0005.*

**FIGURE 5:**
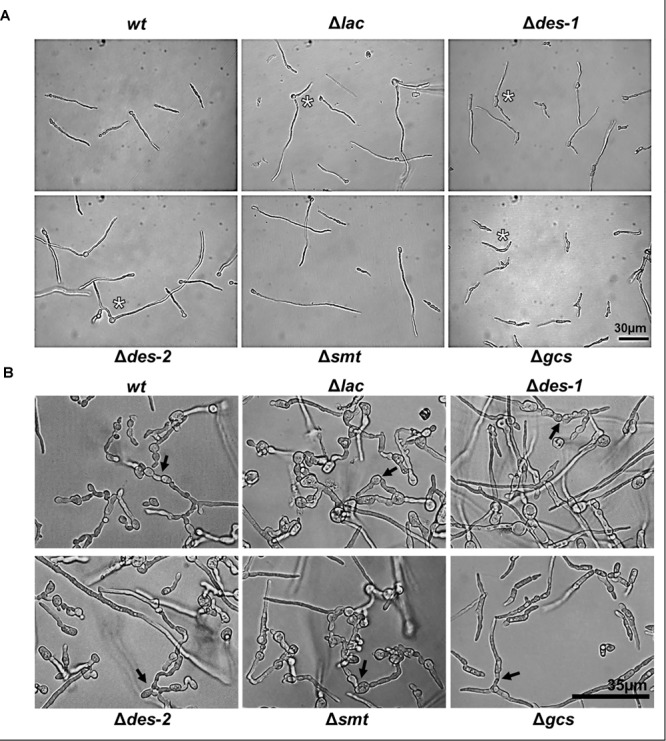
Effect of GlcCer depletion on germination and CAT-fusion of *N. crassa*. **(A)** Phenotypes of 6 h old germlings: 10^4^ conidia/mL were incubated in 0.2 × Vogel’s medium. Asterisks indicate multiple germ tubes in Δ*lac*, Δ*des-1*, Δ*des-2*, and Δ*gcs* mutants. **(B)** CAT-fusion: (5 × 10^5^ conidia/mL were cultivated for 6 h in 0.2 × Vogel’s medium). Black arrows indicate CAT-fusion. Scale bar 30 μm in **(A)**, scale bar 35 μm in **(B)**.

We next determined the phenotype of the mutants in submerse growth conditions. The germination efficiency of all strains was similar (Table 5, control). The germ tube length reached 53.4 ± 6.9% in the control strain and was similar in the mutants, except for the Δ*gcs* strain, which suffered from a significant growth retardation (36.7 ± 2.8%; *p* ≤ 0.05) compared to the control (Table 6 and Figure 5A). The overall dry biomass, however, was similar in all tested strains after 72 h of cultivation, which indicates that the Δ*gcs* mutant is able to overcome this early growth defect (Supplementary Data Sheet S1, Supplementary Figure S7). Notably, all mutants were impaired in polarized germination and exhibited mostly two germ tubes compared to predominantly one in the reference strain (Table 7). No defects in CAT fusion could be observed in any of the strains tested (Figure 5B).

**Table 5:** The effect of GlcCer depletion on the colony establishment of *N. crassa*.

	Germination efficiency [%]^§^		
Strain	Control^a^ untreated	PAF^b^ 0.06 μM	PAFB^b^ 0.25 μM
*wt*	84.1 ± 3.1	85.3 ± 2.1	53.9 ± 4.6^∗∗∗^
Δ*lac*	85.5 ± 2.4	86.7 ± 1.4	49.5 ± 7.2^∗∗∗^
Δ*des-1*	86.1 ± 4.1	85.5 ± 5.1	54.2 ± 3.5^∗∗∗^
Δ*des-2*	89.1 ± 1.3	88.0 ± 2.0	40.4 ± 5.0^∗∗∗^
Δ*smt*	85.9 ± 4.2	85.5 ± 4.0	50.7 ± 7.1^∗∗^
Δ*gcs*	83.9 ± 5.3	84.1 ± 4.5	49.5 ± 8.7^∗∗∗^

*^§^The germination efficiency is indicated in (%) compared to the total conidial counts, which was set to be 100%. All values are given as mean ± SD (*n* = 3). Significant differences (*p*-values) between values were determined by comparison with ^*a*^the reference strain (*wt*) or with ^*b*^the respective untreated strain, respectively. ^∗∗^*p* ≤ 0.005; ^∗∗∗^*p* ≤ 0.0005.*

**Table 6:** The effect of GlcCer depletion on the germ tube length of *N. crassa*.

	Germ tube length [μm]		
Strain	Control^a^	PAF^b^ 0.06 μM	PAFB^b^ 0.25 μM
*wt*	53.4 ± 6.9	44.0 ± 7.3^∗^	17.5 ± 2.1^∗∗∗^
Δ*lac*	61.9 ± 3.9	47.8 ± 13.0	15.7 ± 0.5^∗∗∗^
Δ*des-1*	45.1 ± 20.0	36.7 ± 10.9	16.5 ± 1.2^∗^
Δ*des-2*	52.0 ± 4.0	45.9 ± 1.9	17.6 ± 2.2^∗∗∗^
Δ*smt*	62.0 ± 13.3	54.9 ± 4.0	18.6 ± 1.3^∗∗^
Δ*gcs*	36.7 ± 2.8^∗^	33.0 ± 6.2	14.0 ± 0.7^∗∗∗^

*All values are given as mean ± SD (*n* = 3). Significant differences (*p*-values) between values were determined by comparison with ^*a*^the reference strain (*wt*) or with ^*b*^the respective untreated strain, respectively. ^∗^*p* < 0.05; ^∗∗^*p* ≤ 0.005; ^∗∗∗^*p* ≤ 0.0005.*

**Table 7:** The effect of GlcCer depletion on polarized germination.

Strain	Multiple germ tubes [%]^a^	Single germ tube [%]^a^
*wt*	11.3 ± 3.5	88.8 ± 3.5
Δ*lac*	44.1 ± 3.7^∗∗∗^	55. 9 ± 3.7^∗∗∗^
Δ*des-1*	39.7 ± 2.7^∗∗∗^	60.3 ± 2.7^∗∗∗^
Δ*des-2*	34.0 ± 11.8^∗∗^	66.0 ± 11.8^∗∗^
Δ*smt*	18.7 ± 8.9^∗^	81.3 ± 8.9^∗^
Δ*gcs*	57.4 ± 6.5^∗∗∗^	42.6 ± 6.5^∗∗∗^

*^*a*^The number of conidia with multiple germ tubes vs. only one germ tube is indicated in (%) compared to the total conidial count of the respective strain, which was set to be 100%. Values are given as mean ± SD (*n* = 6). Significant differences (*p*-values) between values were determined by comparison with the reference strain (*wt*). ^∗^*p* ≤ 0.05, ^∗∗^*p* ≤ 0.005, ^∗∗∗^*p* ≤ 0.0005.*

### Defective GlcCer Synthesis Affects Sensitivity to Oxidative Stress

**FIGURE 6:**
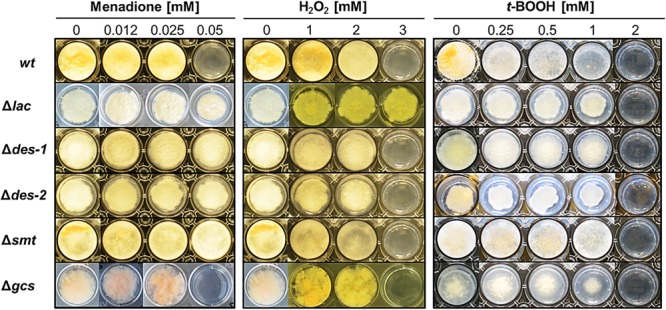
Impact of GlcCer depletion on oxidative stress response. Conidia (10^3^) were point inoculated on Vogel’s agar (0.75%) containing increasing concentrations of oxidative stress inducers menadione (superoxide radical), H_2_O_2_ (hydroxyl radicals) and *tert*-butylhydroperoxide (*t*-BOOH; organic hydroperoxide). Samples were cultivated for 72 h at 25°C.

Ceramide is an important signaling molecule in the induction of ROS and of apoptosis (Ueda, 2015). Depletion in ceramide was referred to lower ROS levels and higher survival rates (Portt et al., 2011). To investigate if the GlcCer synthesis pathway plays a role in oxidative stress response in *N. crassa*, we exposed the mutant strains to increasing concentrations of ROS generators: menadione (superoxide radicals), hydrogen peroxide (H_2_O_2_; hydroxyl radicals) and *tert*-butylhydroperoxide (*t*-BOOH; organic hydroperoxide). Figure 6 shows that the reference strain and the Δ*gcs* mutant tolerated 0.0025 mM menadione, 2 mM H_2_O_2_ and 1 mM *t*-BOOH. The Δ*lac* strain, in contrast, was resistant within the tested concentration ranges of menadione (up to 0.05 mM), H_2_O_2_ (up to 3 mM), but showed the same sensitivity toward *t*-BOOH as the reference strain. The mutants Δ*des-1*, Δ*des-2*, and Δ*smt* were menadione resistant, but H_2_O_2_ and *t*-BOOH sensitive (Figure 6). These results indicate that distinct intermediates of the GlcCer synthesis pathway regulate the sensitivity toward superoxide and hydroxyl radicals but not toward organic hydroperoxides.

### GlcCer Mutants Are Resistant Against PAF but Not PAFB

Next, the GlcCer mutants were tested for their susceptibility to the *P. chrysogenum* AMPs, PAF and PAFB, to investigate whether their antifungal mode of action is regulated by this specific pathway. A concentration dependent growth reduction of the point inoculated *wt* strains on solid medium was observed at a MIC of 1 μM PAF and 4 μM PAFB, respectively (Supplementary Data Sheet S1, Supplementary Figure S8). The growth of the Δ*lac* strain was visibly reduced at 0.5 μM PAF, but reduction was not further aggravated with increasing PAF concentrations (up to 16 μM) (Supplementary Data Sheet S1, Supplementary Figure S9A). The same resistant phenotype within this concentration range was detected for the mutants Δ*des-1*, Δ*des-2*, Δ*smt*, and Δ*gcs* (Supplementary Data Sheet S1, Supplementary Figure S9A). In contrast, a concentration dependent PAFB sensitive phenotype was observed for all mutant strains (Supplementary Data Sheet S1, Supplementary Figure S9B).

**FIGURE 7:**
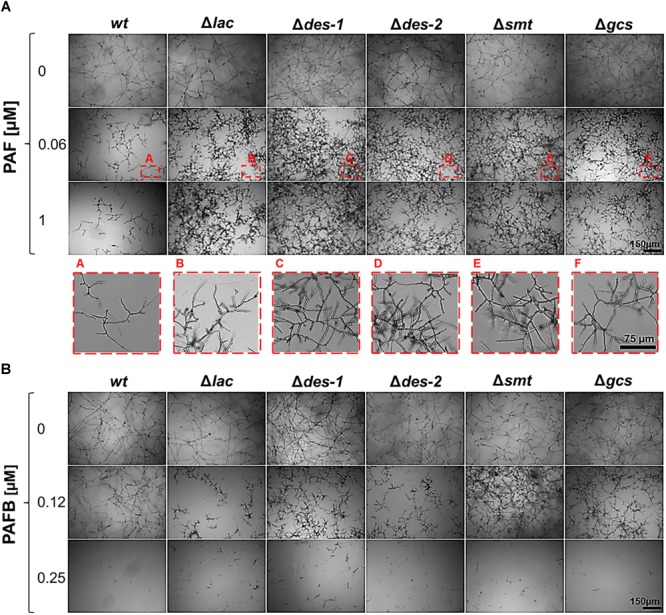
Impact of GlcCer depletion on the AMP susceptibility. **(A)** Sensitivity toward PAF: conidia were grown in the presence of increasing PAF concentrations (0–1 μM) for 30 h. To visualize the mycelial growth at 0.06 μM PAF (corresponding to the MIC of the *wt* strain), magnifications of the insets A–F (framed in red) are presented in the lower panel. **(B)** Sensitivity toward PAFB: conidia were grown in the presence of increasing PAFB concentrations (0–0.25 μM). Scale bar 150 μm and 75 μm.

Broth microdilution assays reflected the results obtained with cultures grown on solid medium. Generally, the growth of *N. crassa* in submerged cultures is inhibited with lower AMP concentrations than on solid media (Huber et al., 2018). The MIC for the *wt* strain was 0.06 μM for PAF and 0.25 μM for PAFB, respectively (Figure 7). All mutant strains proliferated within a PAF concentration range of 0.06–1 μM, though the hyphae were short and hyperbranched as observed in the PAF-treated *wt* (Figure 7A). The germination efficiency of the mutants was similar to that of the *wt* (84.1 ± 3.1%) irrespective of PAF treatment (Table 5) and the germ tube length was only mildly affected - if at all - by PAF (Table 6). In contrast, the mutants were as susceptible to PAFB as the *wt* (MIC 0.25 μM) (Figure 7B). The inhibitory activity of PAFB was also reflected in significantly reduced germination efficiency (Table 5) and significantly shorter germ tubes of all strains tested (Table 6).

### The GlcCer Mutants Are Defective in PAF Interaction

**FIGURE 8:**
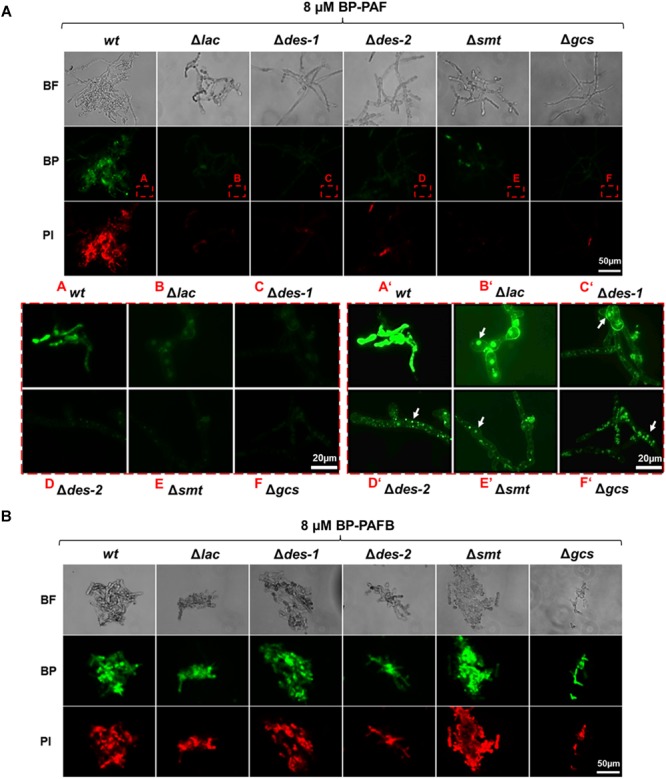
Uptake of BP-PAF and BP-PAFB in germlings of *N. crassa* mutants. Samples were taken after 12 h of incubation of conidia with BP-labeled AMPs. Co-staining with propidium iodide was performed 10 min before imaging. Images were taken with the same exposition time (1500 ms). **(A)** Upper panel: Overview of BP-PAF treated germlings; Red framed lower panel left A–F: Magnification of BP-PAF treated germlings; Framed lower panel right A’–F’: Magnification of BP-PAF treated germlings with increased signal intensity. White arrows indicate BP-PAF uptake into intracellular compartments. **(B)** Overview of BP-PAFB treated samples. BF, Brightfield; BP, BODIPY-labeled proteins; PI, Propidium iodide. Scale bar 50 and 20 μm in **(A)**, scale bar 50 μm in **(B)**.

In previous studies, we reported that the uptake of *P. chrysogenum* AMPs and their cytoplasmic localization in sensitive fungi is a regulated process and triggers fungal cell death (Oberparleiter et al., 2003; Sonderegger et al., 2017; Huber et al., 2018). In this study we investigated, to which extent the GlcCer synthesis pathway regulates the interaction of PAF with *N. crassa*. Since PAFB acted in a GlcCer independent way, we used this AMP as a control. To visualize their cellular localization, the *N. crassa* strains were exposed to both BP labeled AMPs (BP-PAF and BP-PAFB) (Huber et al., 2018). Figure 8 shows that BP-PAF and BP-PAFB were taken up in the reference strain where they localized intracellularly. Co-staining with the cell death dye propidium iodide coincided with positive AMP staining in the treated cells and reflected membrane permeabilization and cell death as reported previously (Huber et al., 2018). Interestingly, no or only a very weak BP-PAF and propidium iodide signal could be observed when mutant strains were treated with BP-PAF (applying the same exposure time as for the reference strain) (Figure 8A). When increasing the signal intensity, the residual BP-PAF signals were localized in cellular compartments but not in the cytoplasm (Figure 8A). In contrast, in all mutant strains a *wt*-like localization of BP-PAFB could be confirmed (Figure 8B).

### *In vitro* Protein-Lipid Interaction Is Electrostatically Driven

Zeta-potential measurements were performed to investigate the interaction between PAF and PAFB with membrane lipids as this method reflects on electrostatic interactions, which presumably plays an important role in the binding of the cationic AMPs with the anionic lipid membranes. Thus, titration of LUVs with AMPs was performed in order to monitor if the accessible surface charge is affected, which in turn influences the Zeta-potential (Domingues et al., 2008). Lipid analysis showed that the various mutant strains mainly differed in the amount of PIs and the amount and nature of ceramides (Supplementary Data Sheet S1, Supplementary Figure S4). In addition to lipid extracts of the *wt*, extracts of Δ*lac* and Δ*gcs* were selected. Besides the lack of glycosylated ceramides both mutants had reduced levels of anionic PIs, whereas the Δ*lac* exhibited markedly increased levels of anionic MIPCs. This difference in lipid composition is reflected in the negative Zeta-potential of the lipid extracts being lowest (-39 mV) for Δ*lac* and highest (-29 mV) for Δ*gcs*. The Zeta-potential for *wt* was in between the lipid extracts of the two mutants (-34 mV).

**FIGURE 9:**
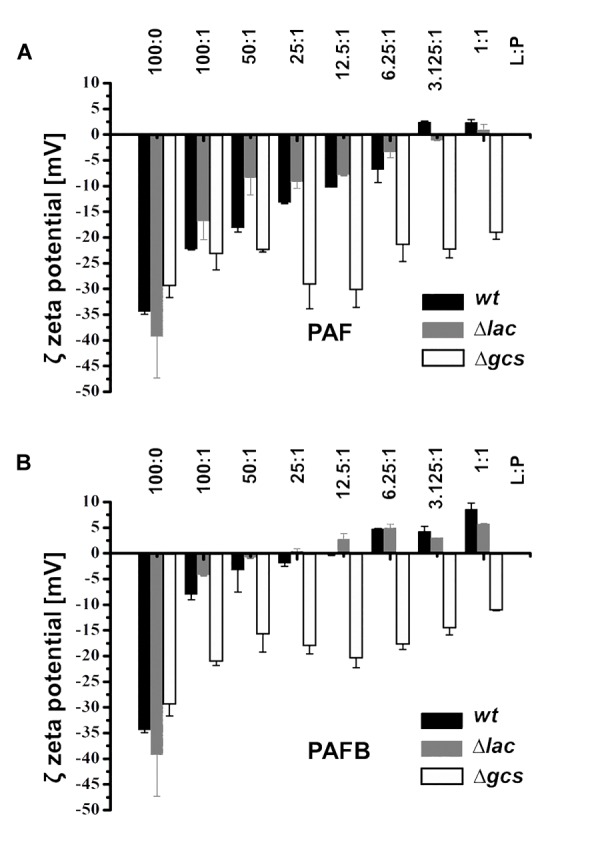
Impact of PAF and PAFB on the surface charge of *N. crassa* lipid vesicles. Zeta potential (mV) of *N. crassa* LUVs obtained from lipid extracts of *wt* (black bars), Δ*lac* (gray bars) and Δ*gcs* (white bars) in the absence and presence of **(A)** PAF and **(B)** PAFB. The AMP concentration was used in relation to 50 μM of LUVs and is expressed as lipid-to-protein molar ratio (L:P). Results are means of 30 measurements in two replicates.

Addition of PAF to the lipid extracts of both *wt* and Δ*lac* progressively altered the Zeta-potential toward a neutral threshold, which was reached at a lipid-to-peptide molar ratio (L:P) of about 3:1 (Figure 9). Adding PAF to lipid extracts of Δ*gcs* only induced a minor change of the Zeta-potential leveling of around -20 mV. In contrast, PAFB induced a dramatic increase of the Zeta-potential to a value of about -5 mV already at a lipid-to-peptide molar ratio of 100:1 leading to neutralization of the surface charge around L:P 25:1 (Figure 9). A further increase of the protein concentration led to an overcompensation of the surface charge. The effect of PAFB on LUVs of Δ*gcs* was less pronounced, but led to a stronger decrease of the Zeta-potential as compared to PAF (-11 vs. -19 mV). In summary, binding of the AMPs to lipid extracts of Δ*lac* was prominent owing to the higher amount of anionic lipids emphasizing the role of electrostatic interactions.

## Discussion

In this study, we carefully investigated the role of the enzymes involved in the GlcCer synthesis pathway in the *N. crassa* membrane lipid composition, growth, conidiation, morphology, stress sensitivity and susceptibility to the *P. chrysogenum* AMPs, PAF and PAFB.

To our best knowledge, this is the first comprehensive report of the contribution of enzymes involved in the GlcCer synthesis pathway to the phenotype of this model fungus. The lack of downstream lipid intermediates and differences in sphingolipid types fully reflected the respective gene deletions in the five analyzed *N. crassa* mutant strains (Δ*lac*, Δ*des-1*, Δ*des-2*, Δ*smt*, Δ*gcs*) and confirmed the identity of the mutants.

Whole-cell lipid extracts analyzed in an untargeted lipidomics approach yielded lipid levels independently of their subcellular location including for example the mitochondria-specific CLs (Schlame and Greenberg, 2017). Extracted features contained all known ceramides and phytoCeramides including their intermediates that were described by Leipelt et al. (2001). Interestingly, phytoCeramides with long chain fatty acids were far less abundant, as were their downstream MIPC products, except for the Δ*lac* strain, where they were slightly accumulated. Non-detectable lipids could still be present in trace amounts at levels below the limit of detection. In all mutant strains as well as the control strains, the phospholipid content as well as its lipid profile remained unchanged and were thus not affected by any of the GlcCer biosynthesis gene deletions.

The determination of GlcCer abundance in null mutant strains provided new insights into this synthesis pathway, which shows in part similarities but also differences to that of other fungi. The mutants Δ*lac*, Δ*des-1*, and Δ*gcs* were completely impaired in GlcCer synthesis, whereas the Δ*des-2* and Δ*smt* mutants exhibited alternative branching of the pathway ending up in the production of the glycosylated sphingolipids, GlcCerOH:36:1 and GlcCerOH:36:3, respectively. Interestingly, such alternative branching patterns have not been described so far in the *A. nidulans* C9-methyltransferase mutant (Δ*smtA*), which produced *wt*-like GlcCer, whereas in the Δ*smtB* mutant increased levels of unmethylated GlcCer have been detected. The *A. nidulans* Δ*sdeA* mutant (lacking the sphingolipid Δ8-desaturase) accumulated C8-saturated and unmethylated GlcCer (Fernandes et al., 2016).

The change in the membrane sphingolipid composition resulted in a growth defect of *N. crassa*: the mutants showed severe growth retardation and disruption of polarized hyphal extension when grown on solid medium. Similar defects have been repeatedly observed in other fungal species defective in ceramide synthase (*A. nidulans, barA1*), sphingolipid Δ8-desaturase (*A. nidulans*, Δ*sdeA*; *C. albicans*, Δ*sld*), C9-methyltransferase (*F. graminearum*, ΔFg*mt2*; *C. albicans*, Δ*mts1*), and GlcCer synthase (*A. nidulans*, Δ*gcsA*; *C. albicans*, Δ*hsx11*) (Li et al., 2006; Oura and Kajiwara, 2008, 2010; Ramamoorthy et al., 2009; Fernandes et al., 2016).

Similar to the *F. graminearum* mutant Δ*BAR1* lacking the ceramide synthase (Rittenour et al., 2011), all *N. crassa* mutants – except for the Δ*gcs* mutant – produced less conidia than the reference strain. Interestingly, all *N. crassa* mutants exhibited a pronounced shift toward multinuclear macroconidia, which suggests the interference of GlcCer synthesis products in conidial development. Specifically, the observation that the mutants Δ*des-2* and Δ*gcs* produced predominantly arthroconidia and little blastoconidia lets us speculate that these enzymatic steps play an important role in conidiophore development and/or in the developmental timeline of conidiation (Springer and Yanofsky, 1989). In contrast, the *A. nidulans* Δ*gcs* suffered from a severe reduction of conidia (Fernandes et al., 2016). Our results thus allow us further to conclude that glycosylated sphingolipids play a variable role in the conidiation efficiency of different fungal species.

Defects in polarized germination, but not in germination efficiency, could be observed in all *N. crassa* mutants, which mostly exhibited two germ tubes compared to predominantly one of the *wt* strain. A slight reduction in the germ tube length of the Δ*gcs* mutant after 6 h of incubation pointed toward a mild defect in germ tube elongation during early growth phase. We conclude that the glycosylation of CerOH:36:3, CerOH:37:2 and CerOH:37:3 supports fast and efficient colony establishment under liquid conditions (Araujo-Palomares et al., 2007), though the GlcCer depletion had no adverse impact on the fungal biomass after longer growth times. The CAT fusion ability in the *N. crassa* mutants was not affected by any of the gene deletions, which also matched a previous report for the *N. crassa* Δ*gcs* mutant (Chu, 2013).

It is well known that ceramides and other sphingolipids play an important role in cell signaling (Epstein and Riezman, 2013), e.g., they are involved in oxidative stress response and cell death mechanisms (Barth et al., 2010). Until now, the contribution of GlcCer in oxidative stress response in fungi has been only sparsely characterized. Our study demonstrates that the GlcCer pathway plays no role in organic hydroperoxide (*t*-BOOH) sensitivity, whereas all *N. crassa* mutants were resistant to superoxide radicals (menadione), except for the Δ*gcs* mutant, which was comparably susceptible to menadione as the *wt*. This indicates that the modification of the ceramide fatty acid rather than the presence of a sugar residue regulates the stress response to menadione. The Δ*lac* mutant, however, was the only strain resistant to H_2_O_2_. This stands in contrast to reports on a respective *C. neoformans* (Δ*cer1*) mutant lacking the ceramide synthase, which developed a hypersensitive phenotype when grown in the presence of H_2_O_2_ (Munshi et al., 2018). Additionally, a connection between MIPCs and oxidative stress has been reported for *S. cerevisiae*: reduced M(IP)_2_C levels rendered yeast cells more resistant to ROS induced by H_2_O_2_ (Aerts et al., 2006). Surprisingly and in contrast to these findings, the amount of MIPCs was increased in the H_2_O_2_ resistant *N. crassa* Δ*lac* mutant. Due to the lack of respective data from other filamentous fungi, we assume that the total loss of GlcCer intermediates differently affects the stress response of *N. crassa* compared to yeasts. This, however, awaits further investigation.

Many reports handled fungal sphingolipids as possible binding targets for plant defensins. *In vitro* experiments indicated that the *Raphanus sativus* antifungal protein 2 (RsAFP2) or the *Pisum sativum* defensin 1 (*Ps*d1) interact with fungal GlcCer (Thevissen et al., 2004; de Medeiros et al., 2014). Defects in GlcCer synthesis were associated with lower susceptibility or even resistance of fungi against certain plant defenins (Ramamoorthy et al., 2007, 2009; Fernandes et al., 2016).

Here we could show that the intermediates and the end products of the GlcCer pathway determine the susceptibility of *N. crassa* toward PAF, but not PAFB. All mutants lacking enzymes in the GlcCer biosynthesis exhibited a PAF-resistant phenotype. The energy-dependent uptake and cytoplasmic localization in PAF/PAFB-exposed sensitive fungi is a prerequisite for antifungal activity as reported previously (Oberparleiter et al., 2003; Sonderegger et al., 2017; Huber et al., 2018). The GlcCer mutants showed a disturbed interaction with PAF resulting in significantly diminished protein internalization. In contrast, we could not detect any GlcCer-dependent resistance toward PAFB and its intracellular localization coincided with cell death in *N. crassa*. This is not impossible, since different mechanisms of action have been also described for plant defensins that share high structural similarities (Cools et al., 2017).

Zeta-potential measurements performed with LUVs composed of lipid extracts derived from *wt*, Δ*lac* and Δ*gcs* emphasize that electrostatic interaction is a driving force for the affinity between the *P. chrysogenum* derived AMPs and lipid membranes. PAFB, exhibiting a higher net positive charge than PAF (+5.2 vs. +4.7, respectively) (Huber et al., 2018; Sonderegger et al., 2018), neutralized the membrane surface charge at much lower concentrations and both AMPs bound more efficiently to LUVs of the Δ*lac* than the *wt* strain. One may speculate that this is due to the increase of anionic MIPC in this mutant, exhibiting a high affinity for PAFB. This in turn could partially compensate for the lack of GlcCerOH:36:3, GlcCerOH:37:2, and GlcCerOH:37:3 and is in line with the uptake of PAFB in the *N. crassa* GlcCer mutants. This finding supports a recent computational modeling approach, which showed that adsorption of the antifungal protein AFP from *Aspergillus giganteus* is mainly driven by electrostatic attraction between the cationic protein and the acidic membrane surface (Utesch et al., 2018). Interestingly, none of the two *P. chrysogenum* AMPs was able to neutralize the negative Zeta-potential of LUVs derived from the mutant Δ*gcs* within the investigated concentration range proposing a role of the sugar moiety for high affinity binding of AMPs as suggested also by Utesch et al. (2018).

Importantly, our study evidences that differences in the GlcCer pathway intermediates and their functional role exist between *N. crassa* and other fungal species. Furthermore, we show that the antifungal mode of action of PAF and PAFB differs despite their considerable sequence and structural similarity. Both AMPs follow diverging underlying molecular mechanisms: a GlcCer-dependent route for PAF and a GlcCer-independent route for PAFB. Our data exclude a direct interaction of PAF with GlcCer or other pathway specific intermediates. Instead, our findings allow us to hypothesize that sphingolipids regulate the membrane attraction and stabilization of PAF and PAFB binding, and suggest the necessity of other interaction molecules, whose presence or activity might be influenced by the GlcCer dependent lipid raft organization in the plasma membrane (Santos et al., 2018).

## Author Contributions

FM, MAK, and JZ conceived, supervised and financed the study. FM, MAK, and KL designed the experiments and edited the manuscript. AH performed membrane lipid extractions, phenotypically characterized the mutants, conducted protein localization studies and analyzed the data. MAK and GO performed the lipidomic profiling of membrane extracts and analyzed the data. AH and LK determined the antimicrobial activity. WS performed scanning electron microscopy. NM and KL performed zeta-potential measurements and analyzed the data. All authors contributed to manuscript writing, and read and approved the submitted version.

## Conflict of Interest Statement

The authors declare that the research was conducted in the absence of any commercial or financial relationships that could be construed as a potential conflict of interest.

## Footnotes

1 www.gimp.org

2 www.broadinstitute.org

3 www.candidagenome.org

4 https://blast.ncbi.nlm.nih.gov/Blast.cgi

5 http://www.uniprot.org/

## Abbreviations

AMP: antimicrobial protein
BP: BODIPY FL-EDA
CAT: conidial anastomosis tubes
CL: cardiolipin
ESI: electrospray ionization
GlcCer: glucosylceramide
H_2_O_2_: hydrogen peroxide
HPLC: high-performance liquid chromatography
(LC-) MS/MS: (liquid chromatography) tandem mass spectrometry
LCB: long chain base
LUV: large unilamellar vesicle
MIC: minimal inhibitory concentration
MIPC: mannosylinositol phosphorylceramides
MS: mass spectrometry
PAF: *Penicillium chrysogenum* antifungal protein
PAFB: *Penicillium chrysogenum* antifungal protein B
PC: phosphatidylcholines
PE: phosphatidylethanolamines
PI: phosphatidylinositols
PT: phosphinothricin
ROS: reactive oxygen species
*t*-BOOH: *tert*-butylhydroperoxide
