# Supplementary material for: Membrane Sphingolipids Regulate the Fitness and Antifungal Protein Susceptibility of Neurospora crassa
Source: Front Microbiol. 2019 Apr 11;10:605. doi: 10.3389/fmicb.2019.00605 (PMC6471014; doi:10.3389/fmicb.2019.00605)
Supplement: Supplementary file 2 [file Data_Sheet_1.pdf]

Supplementary Data Sheet 1

Membrane sphingolipids regulate the fitness and antifungal protein susceptibility of *Neurospora crassa*

Anna Huber[1], Gregor Oemer[2], Nermina Malanovic[3], Karl Lohner[3], Laura Kovács[1], Willi Salvenmoser[4], Johannes Zschocke[2], Markus A. Keller[2*] and Florentine Marx[1*]

[1]Division of Molecular Biology, Biocenter, Medical University of Innsbruck, Innsbruck, Austria; [2]Division of Human Genetics, Medical University of Innsbruck, Innsbruck, Austria; [3]Institute of Molecular Biosciences, Biophysics Division, University Graz, Graz, Austria; [4]Institute of Zoology, University of Innsbruck, Innsbruck, Austria.

Running title: Sphingolipids of *Neurospora crassa*

Key words: sphingolipids, glucosylceramide, lipidomics, *Neurospora crassa*, antimicrobial proteins, *Penicillium chrysogenum*

Supplementary Methods

Verification of GlcCer mutants by PCR

To verify the gene knockout in the mutant strains, a gene specific PCR-reaction was performed. Genomic DNA of all mutant strains was extracted according to Zadra et al. (2000) and used as template for PCR. Gene deletion was accomplished by the replacement of the gene with the hygromycin resistance (*hygR*) cassette (Park et al. 2011). To verify the presence of the *hygR* cassette, we used the primer hygRvf_3f, which binds in the resistance gene *hygR* and the primer LAC-1vf_3r, FGSC#15707_3r, FGSC#16221_3r, FGSC#13992_3r or GCSvf_3r, respectively (Supplementary Table S5). These latter primers bind to the 3'UTR region of the respective gene that was replaced by the resistance marker cassette. For the verification of the site-specific integration of the *hygR* cassette we used the primer pair LAC-1vf_3r and Lac_fw_outs_flank 5′ for the Δ*lac* mutant, the primer pair FGSC#15707_3r and 15707_fw_outs_flank5′ for the Δ*des-1* mutant, FGSC#16221_3r and 16221_fw_outs_flank5′ for the Δ*des-2* mutant, FGSC#13992_3r and 13992_fw_outs_flank5′ for the Δ*smt* mutant and GCSvf_3r and GCS_fw_outs_flank5′ for Δ*gcs* mutant. As controls we included the *wt* and the Δ*ku70* strain.

Northern analysis

N. crassa wt, Δ*ku70* and Δ*smt* were grown in liquid medium overnight at 25°C in shaking flasks and total RNA was extracted from the mycelium using TRI Reagent (Sigma-Aldrich). Ten μg of total RNA per lane were loaded on a 1.2% formaldehyde-agarose gel and blotted onto Hybond-N membranes (Amersham Biosiences). The primer pair Smt northern_fw and Smt northern_rev was used for PCR-based amplification of the *smt* gene specific hybridization probe labelled with digoxigenin (DIG) (Roche) (Supplementary Table S5).

Lipid quantification

For total lipids quantification in extracts, a colorimetric adsorption method was used, modified from Cheng et al. (2011). The assay was performed in 96-well plates. A standard series from olive oil, diluted in chloroform:methanol (2:1), was prepared. Different volumes of the total lipid extracts and the standards were added to the wells in triplicates, respectively and dried for 15 min at 70°C. Subsequently 100 μL 96% H_2SO_4 (v/v) were added and samples were incubated for 20 min at 70°C. After cooling the microplate on ice, sample background adsorption was photometrically determined by measuring the OD_{540} with a Fluostar Omega microplate reader (BMG Labtech). Then 50 μL 17% phosphoric acid (v/v) containing 0.2 mg/mL vanillin were added and after 30 min incubation at 25°C, adsorption was determined again at 540 nm. Based

on the standard series, the lipid content of the extracts was calculated using Microsoft Excel 2010 software (Microsoft Corp.).

Microscopic imaging of *N. crassa* grown on solid medium

Five µL of a 2 × 10^5/mL spore suspension of the *N. crassa wt* and mutant strains were dotted on Vogel´s agar plates and incubated at 25°C. Images of the colony edges were taken after 24 h using an inverted Leica DM IL LED microscope (Leica Microsystems) combined with an AxioCam MR3 camera (Carl Zeiss GmbH) and processed with AxioVision software (Carl Zeiss GmbH).

Electron microscopy

Samples for scanning electron microscopy (SEM) were prepared by dotting 5 µL of a 2 × 10^5/mL spore suspension of the *N. crassa wt* strains and the Δ*lac* and Δ*gcs* mutants on Vogel´s agar. Plates were incubated at 25°C for 72 h under continuous light. Samples were fixed with 2.5% glutaraldehyde in 0.1 M cacodylate buffer (pH 7.3) for 2 h to overnight at 4°C. After washing with buffer, samples were post-fixed with 1% osmium tetroxide in 0.05 M cacodylate buffer for 1-2 h at room temperature and rinsed with buffer. Representative pieces were cut out of the agar, dehydrated in increasing methanol concentrations, critical point dried and gold coated. Samples were examined with a ZEISS DSM 950 scanning electron microscope (Carl Zeiss GmbH) using 15 kV. Images were taken with a Pentax digital camera and gray scales were adjusted using Adobe Photoshop.

Supplementary Tables

Table S1. Media used in this study.

Culture medium	Composition
Minimal medium (MM)	0.3% $NaNO_3$, 0.05% $MgSO_4 \times 7H_2O$, 0.05% KCl, 0.005% $FeSO_4 \times 7H_2O$, 2% D(+)-sucrose (w/v), 2.5% KPO_4-buffer (1M, pH 5.8), 0.1% trace elements A (v/v)
Trace elements A	0.1% $FeSO_4 \times 7H_2O$, 0.9% $ZnSO_4 \times 7H_2O$, 0.04% $CuSO_4 \times 5H_2O$, 0.01% $MnSO_4 \times H_2O$, 0.01% H_3BO_3, 0.01% $Na_2MoO_4 \times 2H_2O$ (w/v)
Vogel´s medium	2.0% salt solution (v/v), 2.0% D(+)-sucrose (w/v)
Salt solution	15.0% sodium citrate, 25.0% KH_2PO_4, 10.0% NH_4NO_3, 1.0% $MgSO_4 \times 7H_2O$, 0.1% $CaCl_2$ (w/v), 0.10% trace elements B, 0.5 ng/mL biotin (w/v)
Trace elements B	5.0% citric acid × H_2O, 5.0% $ZnSO_4 \times 7H_2O$, 0.97% $FeSO_4 \times 7H_2O$, 0.25% $CuSO_4 \times 5H_2O$, 0.05% $MnSO_4 \times H_2O$, 0.05% H_3BO_3, 0.05% $Na_2MoO_4 \times 2H_2O$ (w/v)

Table S2. Equipment and parameters for HPLC and MS/MS analysis.

HPLC: Dionex® Ultimate 3000		MS: Thermo Scientific® LTQ Velos	
Column	Agilent® Poroshell 120 RO EC-C8	Source	Electrospray ionisation Positive mode
Column oven temperature	50°C	Capillary temperature	275°C
Sampler temperature	10°C	Source voltage	3.8 kV
Mobile Phase A	10 mM NH_4COOH + 0.2% CHCOOH in 60/40 Acetonitrile/H_2O	Full MS mass range	460-1650 (m/z)
Mobile Phase B	10 mM NH_4COOH + 0.2% CHCOOH in 90/10 Isopropanol/Acetonitrile	Scan rate/<br>Scan type	Normal/<br>Profile
Flow rate	0.400 ml/min	Activation type	CID
Gradient	40% for 2 min	Isolation width	1.0
	40% to 74% in 20 min	N. Collision energy	38
	74% to 99% in 30 sec	Default charge state	1
	99% for 4 min	Activation time	10 ms
	99%-62% in 30 sec	MS2 mass range	5–105% of m.o.p.
	40% for 1 min	Microscans MS1	3
Sample loading	20 µl loop	Microscans MS2	5
Injection volume	10 µl	Dynamic exclusion	enabled
Wash volume	200 µl	Exclusion duration	8 s

Table S3. List of lipid species features included into the analysis.

Feature Name	m.z	RT (min)	Adduct
PE:28:0 (ISTD)	636.48	2.7	[M+H]+
PE:32:3	686.48	2.5	[M+H]+
PE:32:2	688.49	3.1	[M+H]+
PE:32:1	690.51	3.8	[M+H]+
PE:32:0	692.52	4.7	[M+H]+
PE:34:4	712.49	2.65	[M+H]+
PE:34:3	714.51	3.3	[M+H]+
PE:34:2	716.52	4.05	[M+H]+
PE:34:1	718.54	5	[M+H]+
PE:36:6	736.49	2.3	[M+H]+
PE:36:5	738.51	2.8	[M+H]+
PE:36:4	740.52	3.2	[M+H]+
PE:36:3	742.54	4.2	[M+H]+
PE:36:2	744.55	5.25	[M+H]+
PC:28:0 (ISTD)	678.5	2.55	[M+H]+
PC:32:3	728.52	2.4	[M+H]+
PC:32:2	730.54	2.9	[M+H]+
PC:32:1	732.55	3.65	[M+H]+
PC:34:5	752.52	2.05	[M+H]+
PC:34:4	754.54	2.5	[M+H]+
PC:34:3	756.55	3.05	[M+H]+
PC:34:2	758.57	3.8	[M+H]+
PC:34:1	760.59	4.7	[M+H]+
PC:36:6	778.54	2.15	[M+H]+
PC:36:5	780.55	2.6	[M+H]+
PC:36:4	782.57	3.2	[M+H]+
PC:36:3	784.59	4	[M+H]+
PC:36:2	786.6	5	[M+H]+
PC:36:1	788.62	6.05	[M+H]+
PC:38:6	806.57	2.75	[M+H]+
PC:38:5	808.59	3.35	[M+H]+
PC:38:4	810.6	4.15	[M+H]+

PC:38:3	812.62	5.15	[M+H]+
PC:38:2	814.63	6.25	[M+H]+
PI:34:1	837.51	3.7	[M+H]+
PI:34:2	835.51	2.95	[M+H]+
PI:34:3	833.41	2.4	[M+H]+
PI:36:4	859.51	2.5	[M+H]+
PI:36:5	857.51	2.1	[M+H]+
Cer:36:2	564.54	5.5	[M+H]+
Cer:36:2	546.54	5.5	[M-H2O+H]+
Cer:36:1	566.55	6.1	[M+H]+
Cer:36:1	548.55	6.1	[M-H2O+H]+
dhCer:36:0	568.57	6.7	[M+H]+
dhCer:36:0	550.57	6.7	[M-H2O+H]+
dhCer:35:0	552.55	5.45	[M+H]+
dhCer:35:0	534.55	5.45	[M-H2O+H]+
Cer:37:2	578.5	6	[M+H]+
Cer:37:2	560.5	6	[M-H2O+H]+
CerOH:37:3	592.5	4.9	[M+H]+
CerOH:37:3	574.5	4.9	[M-H2O+H]+
CerOH:37:2	594.5	5.3	[M+H]+
CerOH:37:2	576.5	5.3	[M-H2O+H]+
CerOH:36:1	582.6	5.45	[M+H]+
CerOH:36:1	564.6	5.45	[M-H2O+H]+
GlcCerOH:36:1	744.6	4.65	[M+H]+
GlcCerOH:36:1	726.6	4.65	[M-H2O+H]+
GlcCerOH:37:3	754.62	4	[M+H]+
GlcCerOH:37:3	736.62	4	[M-H2O+H]+
GlcCerOH:37:2	756.63	4.35	[M+H]+
GlcCerOH:37:2	738.63	4.35	[M-H2O+H]+
GlcCerOH:37:1	758.65	4.9	[M+H]+
GlcCerOH:37:1	740.65	4.9	[M-H2O+H]+
GlcCerOH:36:3	778.62	4	[M+H]+
GlcCerOH:36:3	760.62	4	[M-H2O+H]+
GlcCer:37:2	740.63	3.6	[M+H]+
GlcCer:37:2	722.63	3.6	[M-H2O+H]+

GlcCer:34:2	714.63	3	[M+H]+
GlcCer:34:2	696.63	3	[M-H2O+H]+
CertOH:42:0	684.6	9.8	[M+H]+
CertOH:42:0	666.6	9.8	[M-H2O+H]+
CertOH:41:0	670.6	9	[M+H]+
CertOH:41:0	652.6	9	[M-H2O+H]+
CertOH:40:0	656.6	8	[M+H]+
CL:56:0 (ISTD)	1241.85	14.3	[M+H]+
CL:56:0 (ISTD)	1258.88	14.3	[M+Na]+
CL:68:7	1395.95	14.65	[M+H]+
CL:68:7	1412.95	14.65	[M+Na]+
CL:68:6	1398	15.7	[M+H]+
CL:68:6	1415	15.7	[M+Na]+
CL:68:5	1400	16.65	[M+H]+
CL:68:5	1417	16.65	[M+Na]+
CL:68:4	1402	17.65	[M+H]+
CL:68:4	1419	17.65	[M+Na]+
CL:68:3	1404	18.6	[M+H]+
CL:68:3	1421	18.6	[M+Na]+
CL:70:9	1420	13.8	[M+H]+
CL:70:9	1437	13.8	[M+Na]+
CL:70:8	1422	14.85	[M+H]+
CL:70:8	1439	14.85	[M+Na]+
CL:70:7	1424	15.9	[M+H]+
CL:70:7	1441	15.9	[M+Na]+
CL:70:6	1426	16.9	[M+H]+
CL:70:6	1443	16.9	[M+Na]+
CL:70:5	1428	17.9	[M+H]+
CL:70:5	1445	17.9	[M+Na]+
CL:70:4	1430	18.9	[M+H]+
CL:70:4	1447	18.9	[M+Na]+
CL:72:11	1444	12.95	[M+H]+
CL:72:11	1461	12.95	[M+Na]+
CL:72:10	1446	14	[M+H]+
CL:72:10	1463	14	[M+Na]+

CL:72:10	1448	15.05	[M+H]+
CL:72:9	1465	15.05	[M+Na]+
CL:72:8	1450	16.05	[M+H]+
CL:72:8	1467	16.05	[M+Na]+
CL:72:7	1452	17.15	[M+H]+
CL:72:7	1469	17.15	[M+Na]+
CL:72:6	1454	18.1	[M+H]+
CL:72:6	1471	18.1	[M+Na]+
CL:72:5	1456	19.05	[M+H]+
CL:72:5	1473	19.05	[M+Na]+
MIPCdOH:40:0	1028.6	12.2	[M+H]+
MIPCdOH:40:1	1026.6	10.9	[M+H]+
MIPCdOH:40:2	1024.6	9.6	[M+H]+
MIPCdOH:42:0	1056.6	13.6	[M+H]+
MIPCdOH:42:1	1054.6	12.5	[M+H]+
MIPCdOH:42:2	1052.6	11.2	[M+H]+
MIPCdOH:42:3	1050.6	10	[M+H]+
MIPCdOH:42:4	1048.6	8.8	[M+H]+

Table S4. Genes coding for enzymes involved in GlcCer pathway of *N. crassa*.

Gene ID & name	Open reading frame (ORF)	Number of introns	Chromosome	Protein product
NCU02468<br>Δ*lac*	1809 bp	3<br>length: 75, 139, 69 bp	1	509 aa
NCU08927<br>Δ*des-1*	1384 bp	2<br>length: 73, 93 bp	5	406 aa
NCU02408<br>Δ*des-2*	2007 bp	1<br>length: 131 bp	7	625 aa
NCU07859<br>Δ*smt*	1817 bp	3<br>length: 109, 74, 60 bp	3	525 aa
NCU01116<br>Δ*gcs*	1735 bp	1<br>length: 96 bp	5	546 aa

Table S5. Oligonucleotides used in this study for PCR.

Primer	Sequence 5’-3’	Gene	Product length
hygRvf_3f	CGACAGACGTCGCGGTGAGTTCAG	*hyg*	
LAC-1vf_3r	CTCCAGTTGTAGTATGGTGC	Δ*lac*	1576 bp[a]
FGSC#15707_3r	GAATCACGGAATGTACGAGG	Δ*des-1*	1424 bp[a]
FGSC#16221_3r	TGGTAGACGTATGGTGTAGC	Δ*des-2*	1451 bp[a]
FGSC#13992_3r	CTAGAGCCATGATCAACAGC	Δ*smt*	1418 bp[a]
GCSvf_3r	TCTCCTATCCCTCGTGAAGC	Δ*gcs*	1499 bp[a]
Lac_fw_outs_flank 5′	CGTCCATCTTGCCGTCGTAC	Δ*lac*	3696 bp[b]
15707_fw_outs_flank5′	GGGTGACTGGACAAGAAACG	Δ*des-1*	3528 bp[b]
16221_fw_outs_flank5′	GAGCAGGAACTCAAGAAGGC	Δ*des-2*	3736 bp[b]
13992_fw_outs_flank5′	CGTTGGGTAATGGTATGGG	Δ*smt*	3603 bp[b]
GCS_fw_outs_flank5′	GGATGTCAGCAATGTCAACCG	Δ*gcs*	3786 bp[b]
Smt northern_rev	ATGTCGGAGTCGCATAGCGTC	*smt*[c]	
Smt northern_fw	GATCAGGCCATAAGTGAAGCG	*smt*[c]	

[a]PCR product length resulting from combining the *hygR*-specific oligonucleotide hygR_3f with oligonucleotide specific for the 3'UTR region of the replaced gene.
[b]PCR product length resulting from combining the oligonucleotide specific for the 3′UTR of the respective gene with the oligonucleotide specific for the 5′UTR region outside of the replaced gene.
[c]Oligonucleotides used for generating the *smt*-specific DIG-labelled hybridization probe for Northern blot analysis.

Supplementary Figures

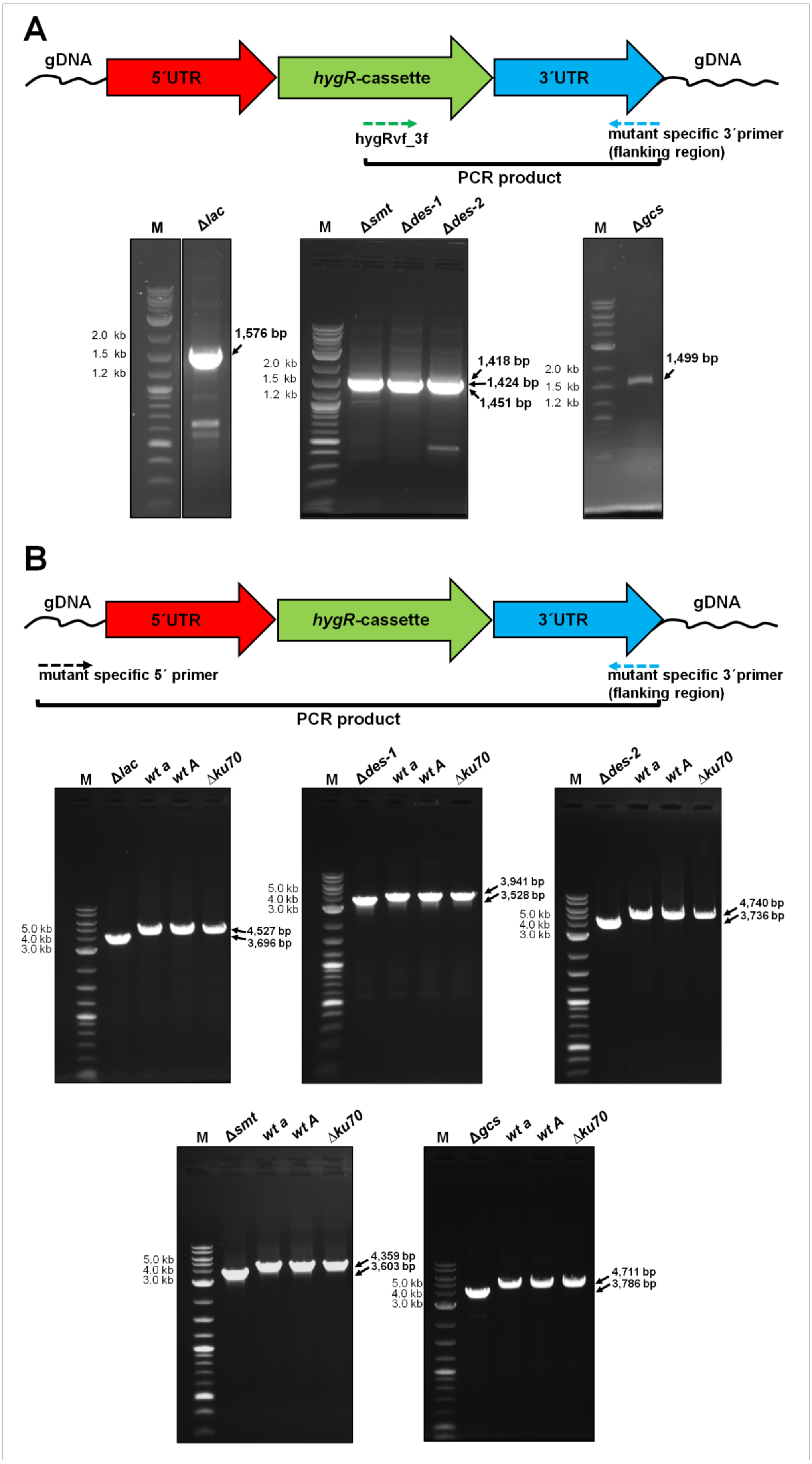

Figure S1. Verification of *N. crassa* knockout mutants with PCR. (A) Top: Scheme to visualize the presence of the *hygR* cassette in the mutants: gene specific 5'-UTR and 3'-UTR region (red and blue arrows) and *hygR* coding sequence (green arrow); hygRvf_3f primer (green dashed arrow) and mutant specific 3'-primers (blue dashed arrow) as listed in Supplementary Table S5; *N. crassa* genome (black wavy lines); PCR product with the expected size (black). **Bottom:** Agarose (1.0 % (w/v)) gel electrophoresis for PCR product analysis. A 2-log size marker (New England Biolabs) was used.

(B) Top: Scheme to visualize the site-specific integration of the *hygR* cassette in the mutants: gene specific 5'-UTR and 3'-UTR region (red and blue arrows) and *hygR* coding sequence (green arrow); mutant specific_5f primers (black dashed arrow) and mutant specific_3r primers (blue dashed arrow) as listed in Supplementary Table S5. The *wt* and the Δ*ku70* strain served as controls. **Bottom:** Agarose (1.0 % (w/v)) gel electrophoresis for PCR product analysis. A 2-log size marker (New England Biolabs) was used.

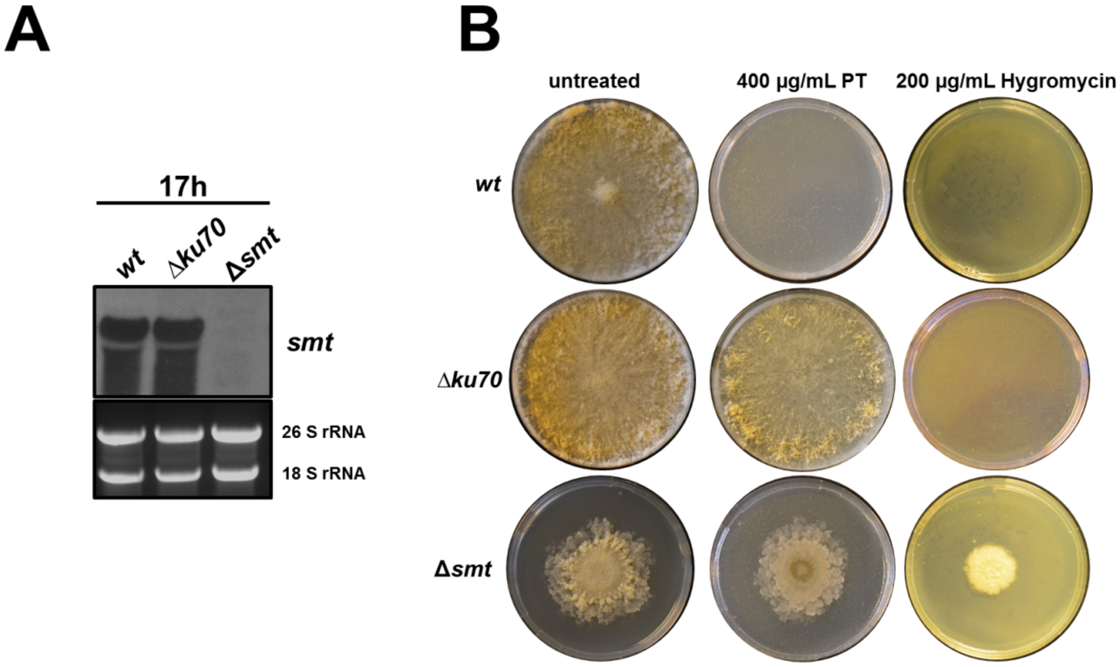

Figure S2. Verification of the *N. crassa smt* deletion strain. (A) Expression of *smt* in 17 h-old submerse cultures of *N. crassa wt, Δku70* and Δ*smt*. Ten µg of total RNA was loaded per lane on a 1.2% denaturing agaraose gel, blotted and hybridized with an *smt*-specific DIG-labelled probe. Ethidium bromide-stained 26S and 18S rRNA is shown as loading control. **(B)** Growth of *N. crassa* strains *wt, Δku70* and Δ*smt* on solid medium supplemented with 400 µg/mL PT or 200 µg/mL hygromycin.

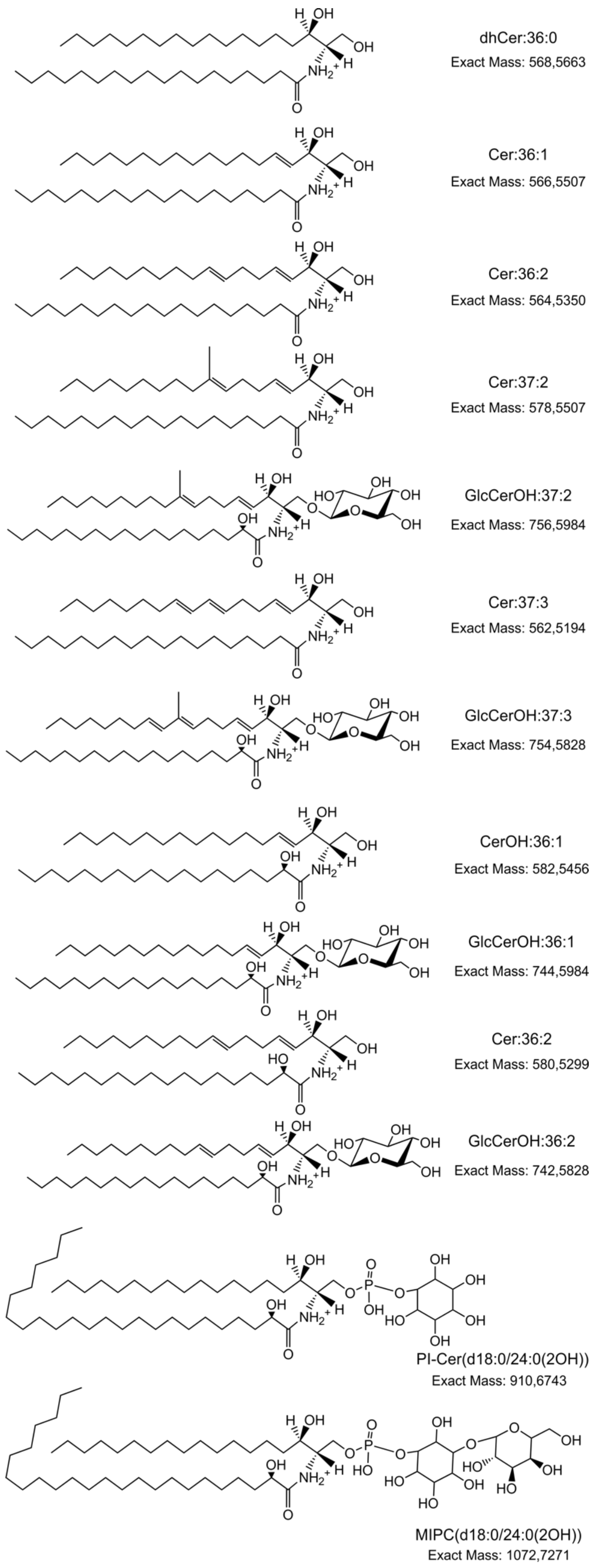

Figure S3. Putative structures of identified ceramide and GlcCer species in *N. crassa*. Structures were plotted with ChemBioDraw (ChemBioOffice).

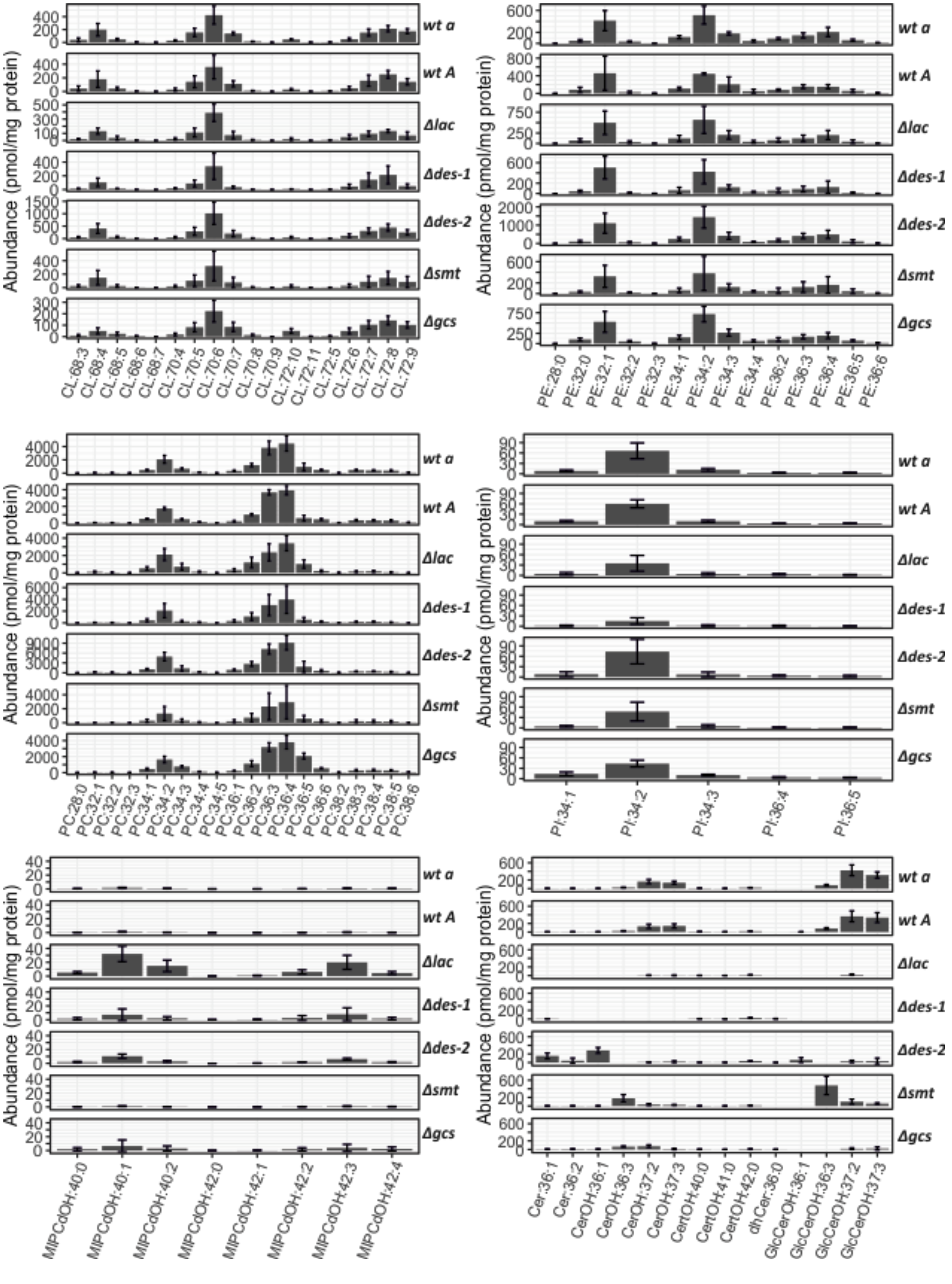

Figure S4. Abundance of ceramide and GlcCer species and other lipid classes in *N. crassa wt* and GlcCer depletion mutants. Phosphatidylcholines (PC), phosphatidylethanolamines (PE), phosphatidylinositols (PI) and cardiolipins (CL), mannosylinositol phosphorylceramides (MIPC), ceramides (Cer), glucosylceramides (GlcCer). Values are given as mean ± SD (n=3).

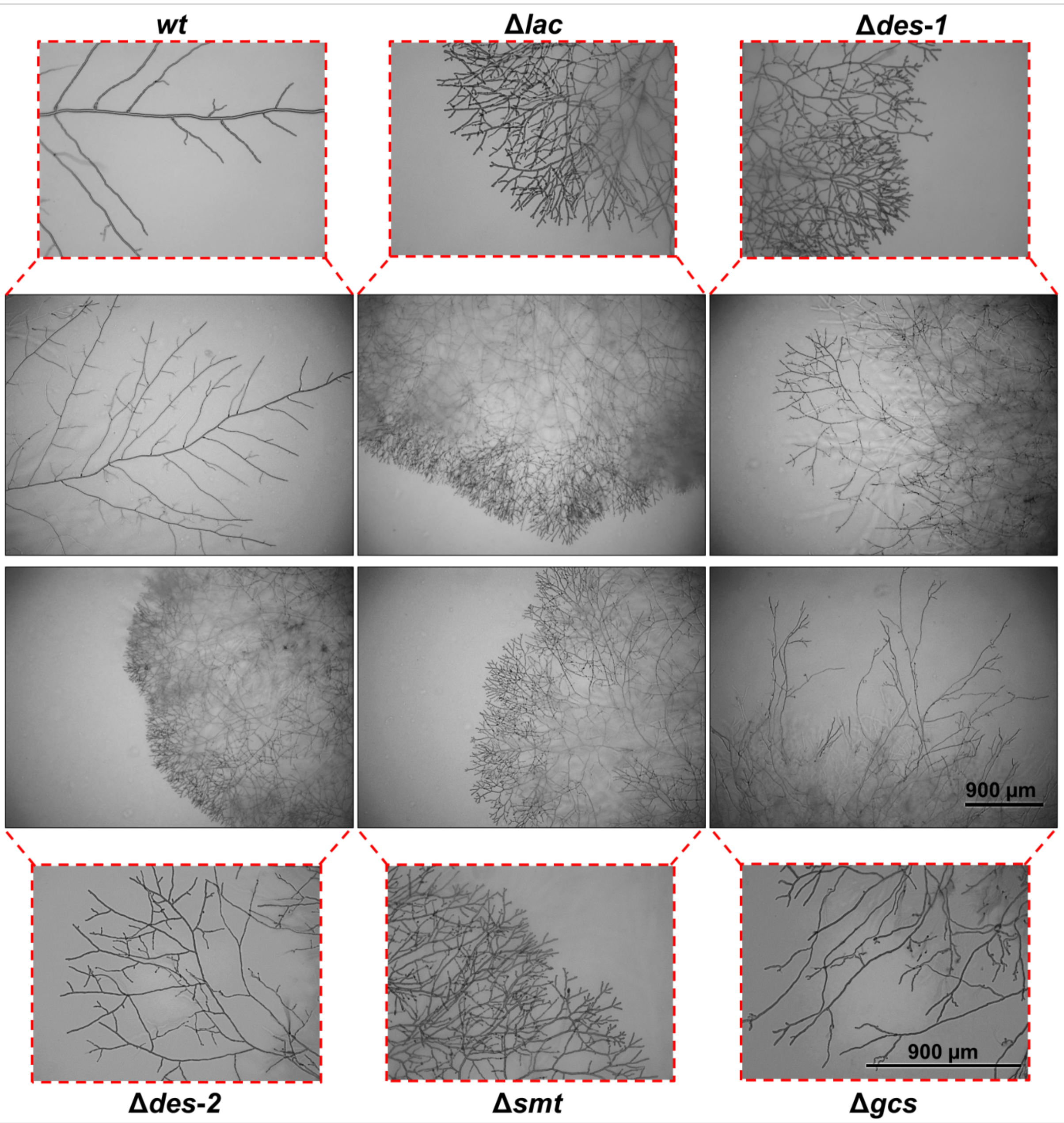

Figure S5. Hyphal morphology of GlcCer depletion strains grown on solid medium. Conidia were point inoculated on Vogel´s agar and grown for 24 h at 25°C before imaging the colony edge. The *wt* served as a reference.

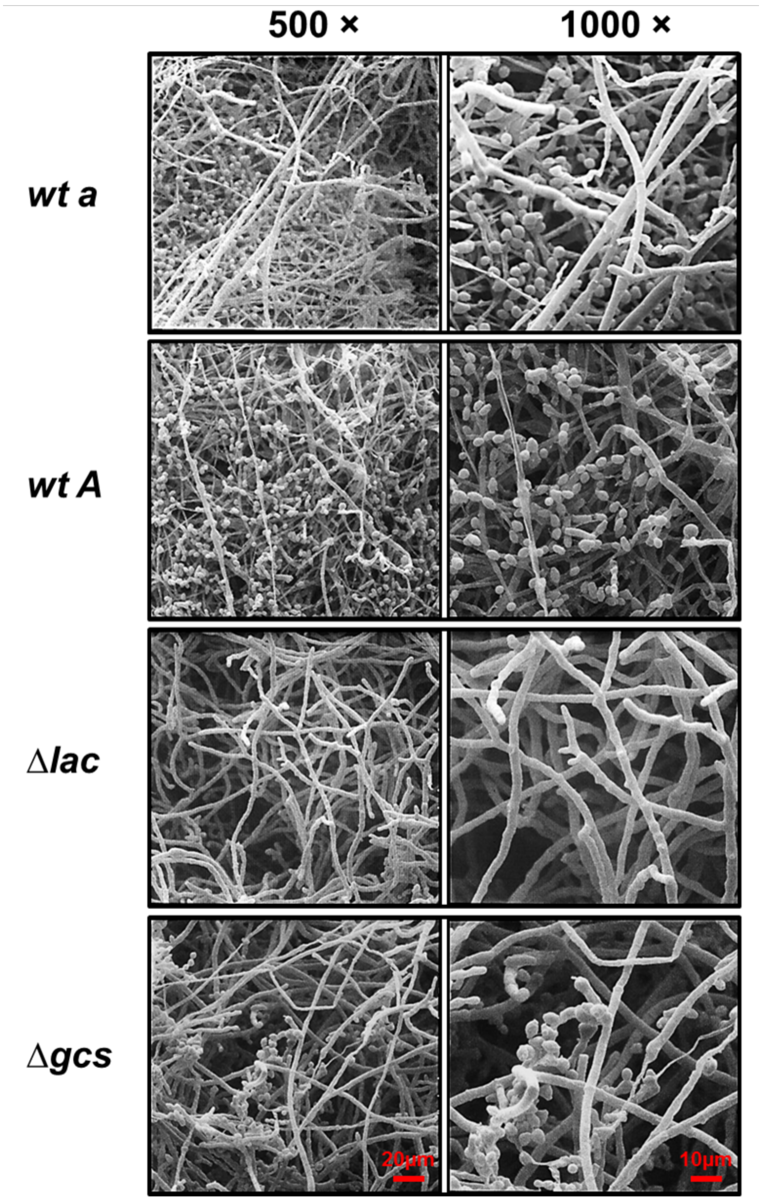

Figure S6. Scanning electron microscopy of *N. crassa wt*, Δ*lac* and Δ*gcs* grown on solid medium. Conidia of the *wt*, Δ*lac* and Δ*gcs* were inoculated on Vogel´s agar and incubated for 72 h at 25°C. Samples were analysed by SEM and aerial hyphae were depicted in magnification of 500 × and 1.000 ×.

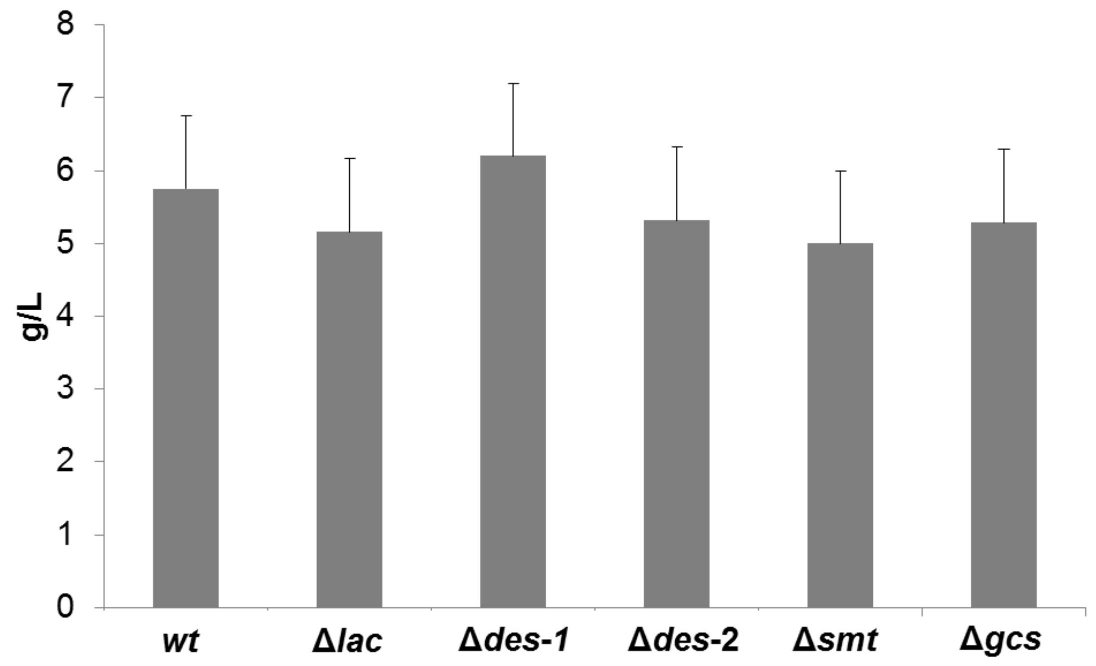

Figure S7. Impact of GlcCer depletion on fungal biomass. Strains were cultivated in liquid Vogel´s medium at 25°C for 72 h. Biomass is indicated as dry weight in gram per liter (g/L). Values are given as mean ± SD (n=3).

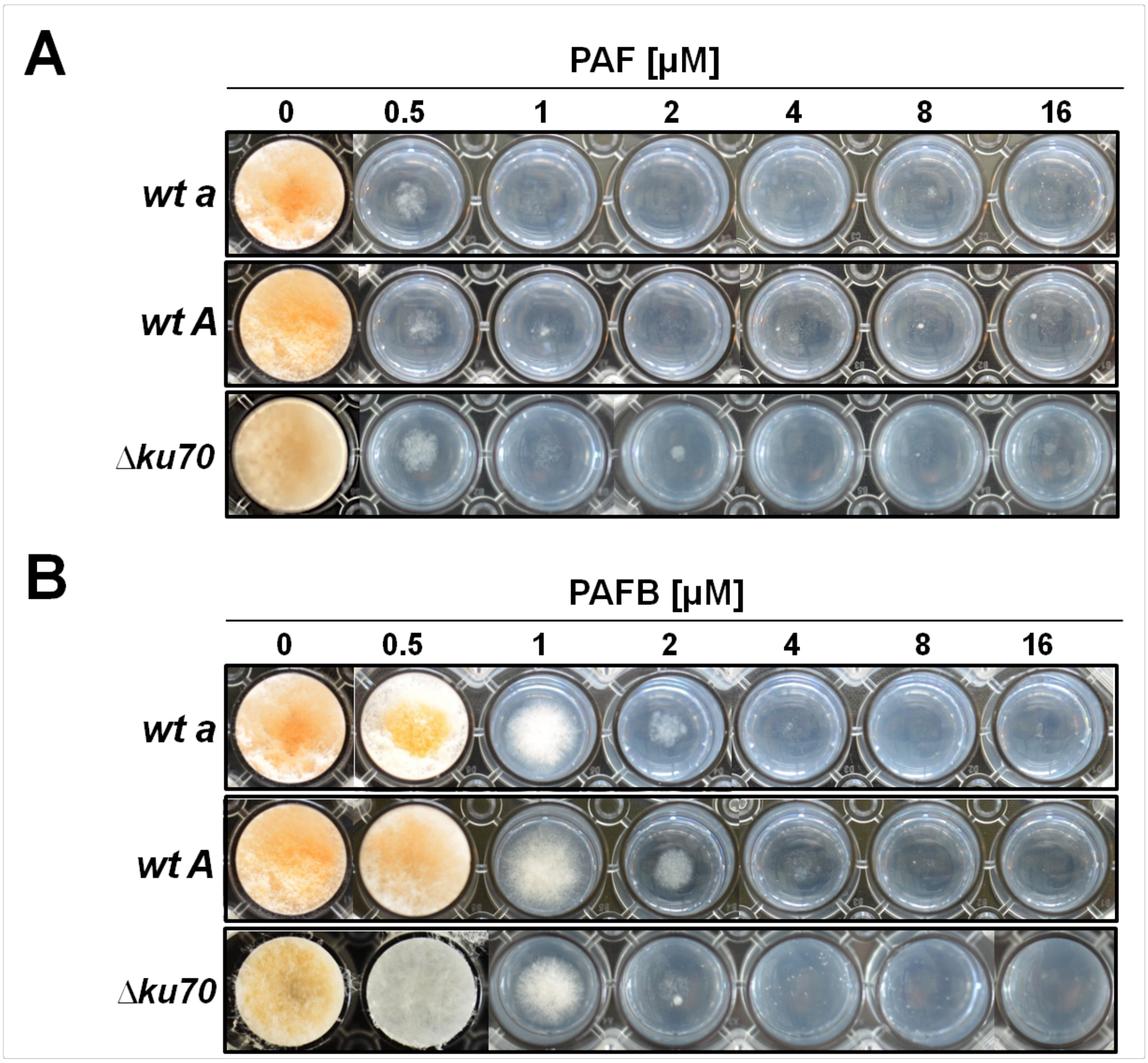

Figure S8. Growth of the *N. crassa wt* and Δ*ku70* strain in the presence of (A) PAF and **(B)** PAFB. Conidia were point inoculated on Vogel´s agar with increasing protein concentrations (0-16 µM) and incubated for 72 h at 25°C.

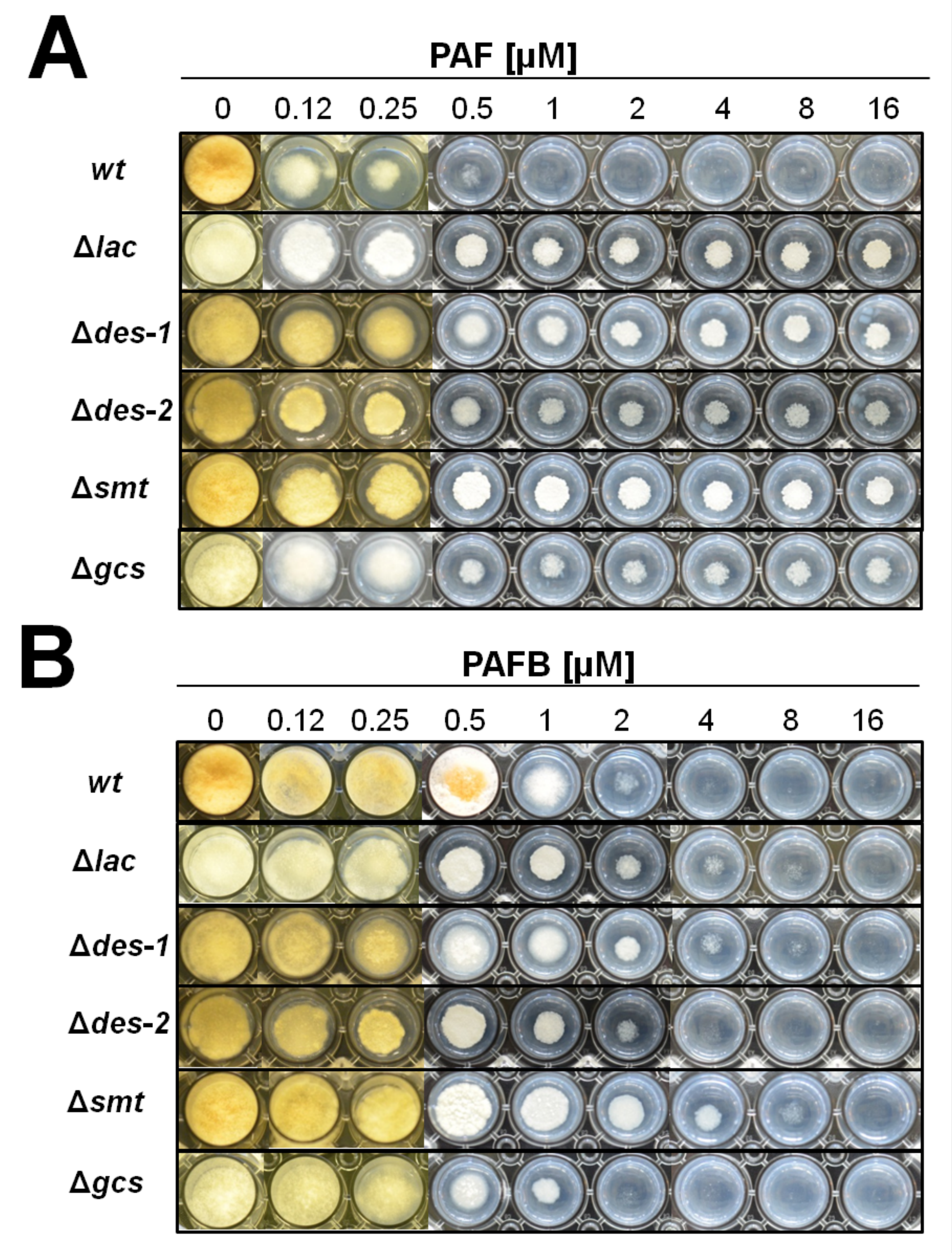

Figure S9. Susceptibility of GlcCer depletion mutants towards PAF and PAFB on solid medium. Conidia were point inoculated in the presence of increasing **(A)** PAF (0-16 µM) or **(B)** PAFB (0-16 µM) concentrations for 30 h at 25°C. The *wt* strain was used as control.

Supplementary References

Cheng, Y. S., Zheng, Y. and VanderGheynst, J. S. (2011). Rapid quantitative analysis of lipids using a colorimetric method in a microplate format. *Lipids* **46**(1): 95-103. doi:10.1007/s11745-010-3494-0.

Park, G., Colot, H. V., Collopy, P. D., Krystofova, S., Crew, C., Ringelberg, C., Litvinkova, L., Altamirano, L., Li, L., Curilla, S., Wang, W., Gorrochotegui-Escalante, N., Dunlap, J. C. and Borkovich, A. (2011). High-throughput production of gene replacement mutants in *Neurospora crassa*. *Methods Mol. Biol.* **722**: 179-189, doi:10.1007/978-1-61779-040-9_13.

Zadra, I., Abt, B., Parson, W. and Haas, H. (2000). *XylP* promoter-based expression system and its use for antisense downregulation of the *Penicillium chrysogenum* nitrogen regulator NRE. *Appl. Environ. Microbiol.* **66**(11): 4810-4816. doi:10.1128/AEM.66.11.4810-4816.2000.
